# Outbreaks of Highly Pathogenic Avian Influenza (H5N6) Virus Subclade 2.3.4.4h in Swans, Xinjiang, Western China, 2020

**DOI:** 10.3201/eid2612.201201

**Published:** 2020-12

**Authors:** Yanbing Li, Minghui Li, Yulei Li, Jingman Tian, Xiaoli Bai, Cen Yang, Jianzhong Shi, Ridengcaicike Ai, Weidong Chen, Wentao Zhang, Jie Li, Yufei Kong, Yuntao Guan, Hualan Chen

**Affiliations:** State Key Laboratory of Veterinary Biotechnology, Harbin Veterinary Research Institute, Chinese Academy of Agricultural Sciences, Harbin, Heilongjiang, China (Y. Li, M. Li, Y. Li, J. Tian, X. Bai, J. Shi, C. Yang, Y. Kong, Y. Guan, H. Chen);; Preventive and Control Center for Animal Disease of Xinjiang Uyghur Autonomous Region, Urumqi, Xinjiang Wei Autonomous Region, China (R. Ai, J. Li);; Preventive and Control Center for Animal Disease of Xinjiang Crops, Urumqi (W. Zhang, J. Li)

**Keywords:** subclade 2.3.4.4h, H5N6, highly pathogenic avian influenza virus, phylogeny, antigenicity, virulence, viruses, respiratory infections, zoonoses, China, bird flu, swans

## Abstract

In January 2020, the subclade 2.3.4.4h of highly pathogenic avian influenza (H5N6) virus infected migratory whooper swans and mute swans in Xinjiang, western China. The virus is lethal to chickens and ducks but has low pathogenicity in mice. Antigenically, this subclade is similar to the H5N1 vaccine seed virus Re-11.

The H5 highly pathogenic avian influenza viruses (HPAIVs) of clade 2.3.4.4 are of great concern because of their global spread and circulation. Ample evidence indicates that clade 2.3.4.4 H5 viruses derived neuraminidase (NA) gene from other low-pathogenicity avian influenza viruses (LPAIVs) co-circulating in migratory birds, and new subtypes of H5N2, H5N5, H5N6, and H5N8 HPAIVs have been detected in wild bird species and poultry globally (*1*,*2*). To date, H5 viruses of clade 2.3.4.4 have evolved into 8 subclades (2.3.4.4a to 2.3.4.4h) according to the World Health Organization’s (WHO) nomenclature system (*1*). Among them, H5N6 is the only subtype that has caused human infections. As of August 2019, a total of 24 human cases have been reported to WHO; the mortality rate is 67% (*3*,*4*).

H5N6 virus of subclade 2.3.4.4a was first detected in poultry in Laos in 2013, then spread to Vietnam and China and caused numerous cases in these areas. H5N8 virus of subclade 2.3.4.4b caused disease outbreaks in wild birds and poultry in Korea in 2014, then spread to North America through bird migration and established a new subclade, 2.3.2.4c. When the H5N8 virus of subclade 2.3.4.4b landed in Europe and Africa, it reassorted with the local LPAIV and produced H5N6 with a novel internal gene cassette in 2017 (*5*). Simultaneously, the H5N6 viruses of subclades 2.3.4.4d, 2.3.4.4e, 2.3.4.4f, 2.3.4.4g, and 2.3.4.4h established in poultry and wild birds in Southeast Asia (*1*,*6*–*8*). Among the 8 subclades of 2.3.4.4, only 3 (H5N6 2.3.4.4b, 2.3.4.4e, and 2.3.4.4f) had been previously detected in swans (*1*).

Since 2004, different vaccines have been developed and widely administered among poultry flocks in China and other countries for H5 avian influenza control, and the vaccine seed viruses used in China have been updated regularly to ensure antigenic match between the vaccine strain and the prevalent strains (*9*,*10*). After the H7N9 HPAIVs emerged in China in 2017, an H5/H7 combined inactivated vaccine was developed and used in poultry (*11*,*12*). Currently, the vaccine seed virus Re-11 is being used to control the clade 2.3.4.4 viruses (*10*). In our study, we analyzed the genetic evolution, antigenicity, and pathogenicity of the H5N6 HPAIVs isolated from migratory whooper swans (*Cygnus cygnus*) and mute swans (*C. olor*) in Xinjiang, western China, in January 2020.

## The Study

**Figure 1:**
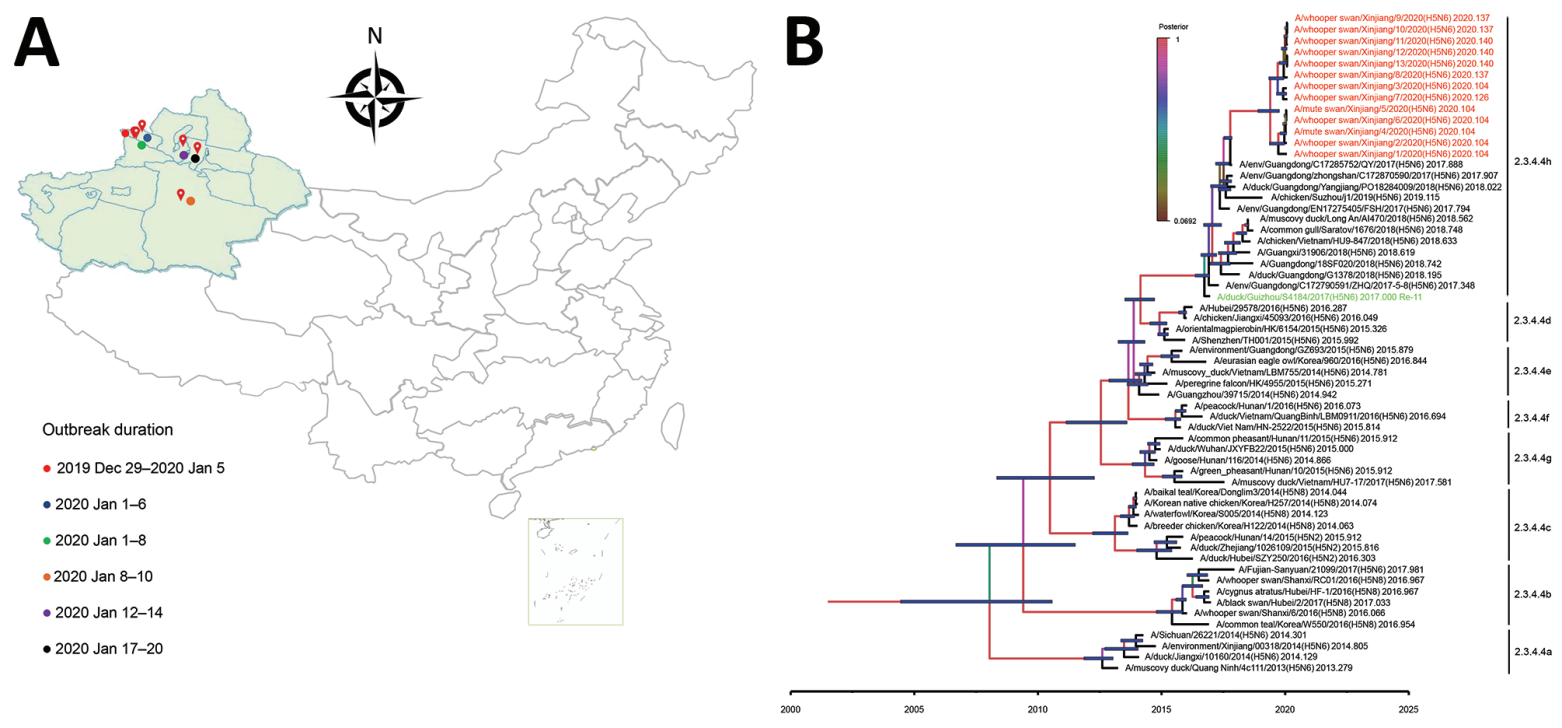
Geography and phylogeny of avian influenza (H5N6) outbreaks among migratory whooper swans (*Cygnus cygnus*) and mute swans (*C. olor*), Xinjiang Province, China, January 2020. A) Disease outbreak sites are marked with red drops, and dates of the outbreaks are indicated. Inset map shows islands in the South China Sea. B) Phylogenetic tree of the hemagglutinin (HA) genes of H5 viruses. The HA gene maximum clade credibility tree of the H5 viruses was constructed by using the BEAST 1.8.4 software package (https://beast-dev.github.io/beast-mcmc). Node bars indicate 95% highest posterior density of the node height. Each branch is colored by posterior probability: the 13 H5N6 viruses reported in this study are shown in red and the HA donor of the H5N1 vaccine Re-11 in green. The time to the most recent common ancestor is labeled at the bottom of the tree, which was estimated by using the Bayesian Markov chain Monte Carlo method in the BEAST 1.8.4 software package.

The first sick whooper swan was found on December 29, 2019, in Sala Village, Samuyuzi Township, Yining City, Xinjiang Uyghur Autonomous Region. The bird died on January 1, 2020. By January 17, deaths had been reported in 58 swans in 6 locations (Table 1; Figure 1, panel A). We received 5 batches of clinical samples from 13 dead birds (11 whooper swans and 2 mute swans), and 13 H5N6 viruses were isolated. The hemagglutinin (HA) subtypes were identified by a hemagglutinin-inhibition test with a panel of H1–H16 subtype antisera, whereas the NA subtypes were detected by reverse transcription PCR with a panel of N1–N9 subtype-specific primers (*11*).

**Table 1:** Avian influenza (H5N6) outbreaks among migratory whooping swans (*Cygnus cygnus*) and mute swans (*C. olor*), Xinjiang Province, China, January 2020

Time	Location description	Bird information		
		Flock size*	No. swans†	
			Total	Dead
2019 Dec 29 to 2020 Jan 5	Small lake in Yining County, Ili Kazak City	>100	100	10
2020 Jan 1–6	Natural park in Yining County, Ili Kazak City	>2,300	40 (270)	6 (3)
2020 Jan 1–8	Natural park in Bole County, Botorla City	160	55	6
2020 Jan 8–10	Natural park in Hejing County, Bayingola City	1150	150	1
2020 Jan 12–14	Wetland in Manas County, Changji City	2,000	800	13
2020 Jan 17–20	Water reservoir in Maguan Chu County, Shihezi City	1,000	150	19

*Estimated number of total migratory birds at that location. †Numbers are whooper swans, except numbers in parentheses, which are mute swans.

To trace the origin of the viruses and understand their genetic relationship, we sequenced the genome of the 13 viruses and performed comparative phylogenetic analysis with the representative H5 HPAIVs that were recommended by WHO (*1*). All 13 H5N6 viruses possess high identity with each other (99.5%–100%); 7 of 8 segments are closely related to the H5N6 virus isolated from environmental samples in Guangdong Province in 2017, whereas the other 1, nonstructural protein gene, is closest to A/chicken/Nghe An/01VTC/2018(H5N6) (Appendix Table 1). The HA gene has the typical highly pathogenic amino acid sequence -RRKR- in its cleavage site, and a few mammalian adaptation mutations were detected in the genome (Table 2) (*13*). In the maximum clade credibility tree, the HA genes of the 13 H5N6 viruses are grouped into subclade 2.3.4.4h with the HA genes of the strains recently found in Vietnam, China, and Russia (*1*) (Figure 1, panel B). The neighbor-joining phylogenic trees of the 8 gene segments are shown in Appendix Figure 1.

**Table 2:** Virulence related molecular markers detected in the WS/XJ/1/2020 (H5N6) virus detected among migratory whooper swans (*Cygnus cygnus*) and mute swans (*C. olor*), Xinjiang Province, China, January 2020

Protein	Amino acid/motif	Phenotypic consequences
Hemagglutinin	Cleavage site motif: -RRKR¯G-	Polybasic cleavage motif sequence required for high pathogenicity of avian influenza viruses in chickens
Neuraminidase	Stalk deletion 58–68	Increased virulence in mice
Polymerase acidic protein	515T	Increased polymerase activity in mammalian cells
Matrix protein 1	30D	Increased virulence in mice
	215A	Increased virulence in mice
Nonstructural protein 1	80–84 deletion	Increased virulence in mice
	42S	Increased virulence in mice
	98F	Increased virulence in mice
	101M	Increased virulence in mice
	222–225 ESEV (PDZ domain)	Increased virulence in mice

The hemagglutinin-inhibition test was performed with polyclonal antiserum generated from the SW/XJ/1/2020(H5N6) and the currently used H5N1 inactivated vaccine Re-11, which carries the HA gene from A/duck/Guizhou/S4184/2017(H5N6) virus (*10*). We found that the SW/XJ/1/2020(H5N6) cross-reacted well with Re-11 antisera, and vice versa (Appendix Table 2), yielding a cross-reactivity R value of 0.26.

We conducted an intravenous pathogenicity index test in chickens with the index virus, WS/XJ/1/2020(H5N6), by following the protocol of the World Organisation for Animal Health (OIE) (*14*). Ten 6-week-old specific-pathogen–free chickens were inoculated with 0.2 mL of virus intravenously, and all the birds died within 3 days postinoculation, yielding an intravenous pathogenicity index test value of 2.59.

**Figure 2:**
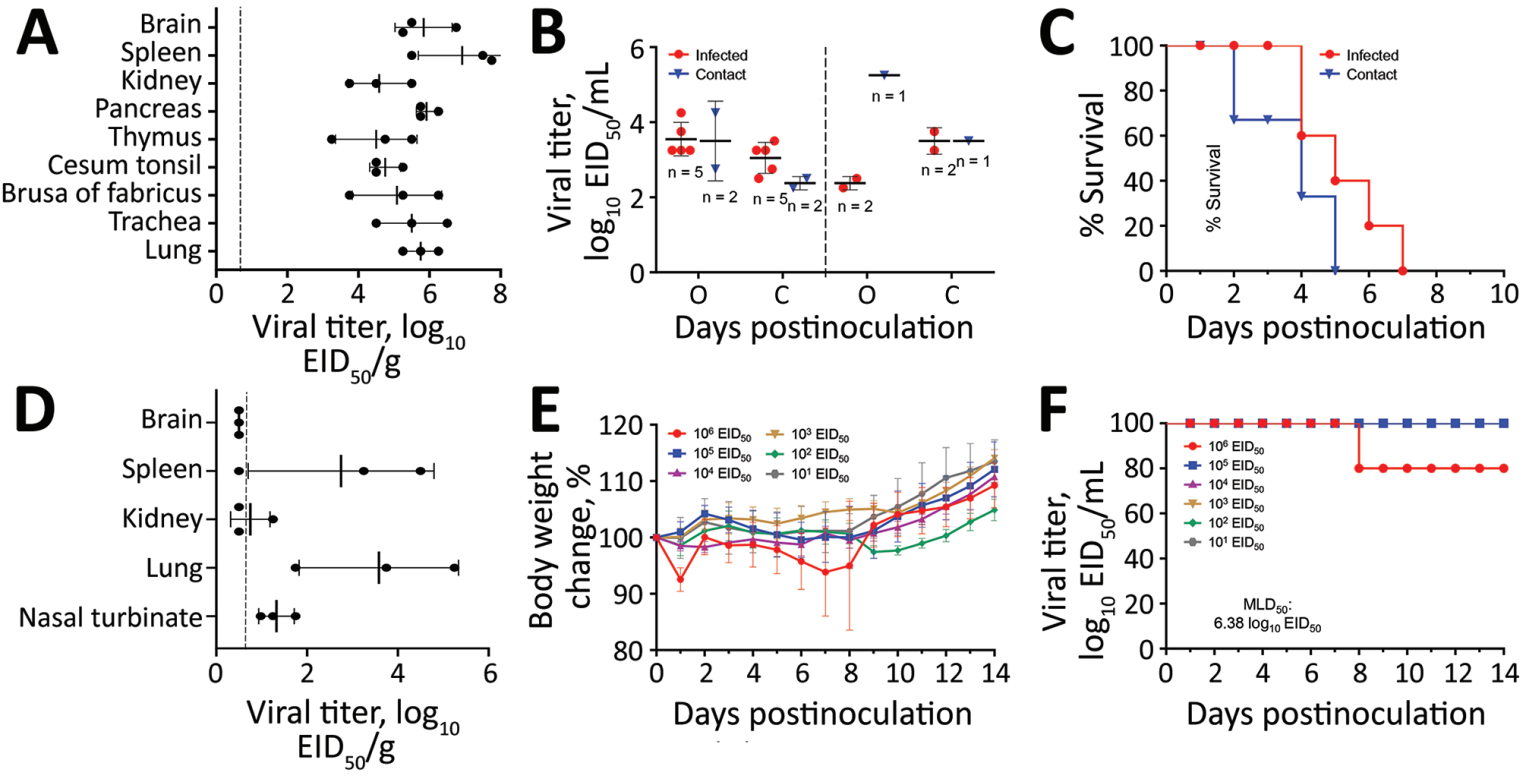
Replication and virulence of the WS/XJ/1/2020(H5N6) isolate in ducks and mice in a laboratory test performed after H5N6 avian influenza (H5N6) outbreaks among migratory whooper swans (*Cygnus cygnus*), Xinjiang Province, China, January 2020. A) Viral titer in organs of ducks that were euthanized on day 3 postinoculation. B) Viral titers in oropharyngeal and cloacal swabs from all surviving ducks were collected on days 3 and 5 postinoculation. C) Lethality of the virus in ducks. D) Viral titer in organs of mice that were euthanized on day 3 postinoculation. E) Bodyweight change of mice after inoculation with different doses of the virus. F) MLD_50_ of the virus. Viral titers in panels A, B, and D are shown as the mean + SD. The dashed lines indicate the lower limit of detection. EID_50_, 50% egg infective dose; MLD_50_, 50% mouse lethal dose.

We tested the virulence and transmission of the WS/XJ/1/2020(H5N6) in ducks as previously described (*2*). Eight 3-week-old specific-pathogen–free ducks were intranasally inoculated with 10^6^ 50% egg infective dose (EID_50_) WS/XJ/1/2020(H5N6), and 3 uninfected ducks were put in the same cage 24 hours later for monitoring transmission. Three virus-inoculated ducks were euthanized on day 3 postinoculation, and high titers of virus were detected in the tested organs (Figure 2, panel A). Virus was also detected in the oropharyngeal and cloacal swabs of the surviving virus-inoculated ducks and the contact ducks on days 3 and 5 postinoculation (Figure 2, panel B). All 5 virus-inoculated ducks and 3 contact ducks died within 7 days postinoculation (Figure 2, panel C).

The replication and 50% mouse lethal dose (MLD_50_) of the WS/XJ/1/2020(H5N6) were evaluated in BALB/c mice as previously reported (*2*). Three mice were intranasally inoculated with 10^6^ EID_50_ of WS/XJ/1/2020(H5N6) in a volume of 50 µL and were euthanized on day 3 postinoculation to assess virus replication in organs, and we found the virus in the brain of 1 mouse, the spleens of 2 mice, and the nasal turbinates and lungs of all 3 mice, but not in the kidneys of any mouse (Figure 2, panel D). To test the MLD_50_, groups of five 6-week-old mice were intranasally inoculated with 10^1^ to 10^6^ EID_50_ of WS/XJ/1/2020(H5N6) in a volume of 50 µL and were monitored for bodyweight loss and death for 14 days. Only 1 of 5 mice that received the highest dose of 10^6^ EID_50_ died on day 8 postinoculation; all other mice survived the 14-day observation period, yielding an MLD_50_ value of 6.38 log_10_ EID_50_ (Figure 2, panel E, F).

## Conclusions

A total of 58 swans died from H5N6 virus infection in 6 wild bird habitats in Xinjiang in January 2020, and we isolated 13 similar H5N6 HPAIVs from the swan specimens. These viruses bear the HAs of subclade 2.3.4.4h, which were previously detected in other bird species but not in swans.

The WS/XJ/1/2020(H5N6) is highly pathogenic to chickens and ducks, and antigenically close to the H5N1 vaccine seed virus Re-11. Although the virus is low pathogenic in mice, it bears multiple residues that can increase its virulence in mammals, and thus might pose a potential threat to public health.

Wild birds carry and spread the H5 HPAIV, as evidenced by the dissemination of the clade 2.2 viruses from Asia to Europe and Africa in 2005, and the intercontinental distribution of the clade 2.3.4.4b viruses in 2014 (*5*,*15*). The prospect of these H5N6 viruses detected in swans being distributed widely by wild birds is worrisome. Therefore, with the migratory season coming, surveillance and preventive measures should be implemented in poultry raised on the migration routes of wild birds.

Appendix Additional information about outbreaks of H5N6 highly pathogenic avian influenza virus subclade 2.3.4.4h in swans, Xinjiang, western China, 2020.
