# Supplementary material for: Outbreaks of Highly Pathogenic Avian Influenza (H5N6) Virus Subclade 2.3.4.4h in Swans, Xinjiang, Western China, 2020
Source: Emerg Infect Dis. 2020 Dec;26(12):2956–60. doi: 10.3201/eid2612.201201 (PMC7706961; doi:10.3201/eid2612.201201)
Supplement: Appendix — Additional information about outbreaks of H5N6 highly pathogenic avian influenza virus subclade 2.3.4.4h in swans, Xinjiang, western China, 2020. [file 20-1201-Techapp-s1.pdf]

Article DOI: https://doi.org/10.3201/eid2612.201201

Outbreaks of Highly Pathogenic Avian Influenza (H5N6) Virus Subclade 2.3.4.4h in Swans, Xinjiang, Western China, 2020

Appendix

Biosafety Statement and Facility

The diagnosis of H5N6 and all experiments with live H5N6 viruses were conducted in the enhanced animal biosafety level 3 (ABSL3+) facility in the HVRI of the CAAS, which is approved for such use by the Ministry of Agriculture and Rural Affairs of China. All animal studies were approved by the Review Board of the HVRI, CAAS.

Animal Testing

All experiments using animals were carried out in strict accordance with the recommendations in the Guide for the Care and Use of Laboratory Animals of the Ministry of Science and Technology of the People’s Republic of China. All protocols were approved by the Committee on the Ethics of Animal Experiments of the HVRI of the CAAS.

SPF Eggs

SPF embryonated chicken eggs (CEEs) were obtained from Harbin Weike Biotechnology Development Company, Harbin, China.

Chickens and Ducks

Six-week-old specific-pathogen-free (SPF) chickens (White Leghorn) and 3-week-old SPF ducks (Shaoxin shelduck, a local bred) were obtained from the Experimental Animal Division of HVRI and housed in ventilated isolators (maximum 8 birds per isolator) in the enhanced animal biosafety level 3 (ABSL3+) facility at the HVRI of the CAAS.

Mice

Five-week-old female BALB/c mice were purchased from Vital River Laboratories (Beijing, China) and housed in ventilated cages (maximum 8 mice per cage) in the enhanced animal biosafety level 3 (ABSL3+) facility at the Harbin Veterinary Research Institute (HVRI) of the Chinese Academy of Agricultural Sciences (CAAS).

Virus Isolation and Identification

The 13 H5N6 viruses used in this study were isolated from samples of dead wild birds sent to the National Avian Influenza Reference Laboratory for the diagnosis of suspected cases of influenza A virus infection in January, 2020 (Table 1, https://wwwnc.cdc.gov/EID/article/26/12/20-1201-T1.htm. The samples were inoculated in 9–11 days SPF CEEs, identification of the HA subtype in the allantoic fluid of HA positive chicken eggs was performed using the hemagglutinin inhibition (HI) test with a panel of H1-H16 reference sera, while the NA subtype was verified by RT-PCR analysis using a panel of N1-N9 subtype primers (*1*,*2*).

Genome Sequencing and Phylogenetic Analysis

The RNA of influenza A(H5N6) viruses were extracted from the allantoic fluid of virus-infected chicken eggs by using the QIAmp viral RNA mini kit (Qiagen, Hilden, Germany). Reverse transcription PCR was performed with a set of gene-specific primers, and the products were sequenced by using an Applied Biosystems DNA analyzer. Primer sequences are available upon request. The nucleotide sequences were edited by using the Seqman module of the DNAStar package. Phylogenetic analysis was performed by employing the neighbor-joining method by using the Mega 6.0.6 ClustalW software package. The tree topology was evaluated by 1,000 bootstrap analyses, and a 97% sequence identity cutoff was used to categorize the groups of each gene segment in the phylogenetic trees (*3*).

A Bayesian time-resolved phylogenetic tree was also created for the hemagglutinin gene of group 1 and 2 viruses using BEAST 1.8.4 (*4*). The SRD06 nt substitution model, the uncorrelated relaxed clock with a log-normal distribution, and the Skygrid flexible effective

population-size tree prior were selected for the analysis. A Markov Chain Monte Carlo (MCMC) chain was run for 30,000,000 steps, with sampling every 3,000 steps. The first 10% of the samples were discarded as burn-in. The above MCMC settings were chosen to achieve a post burn-in effective sample size of at least 200 in all parameters, as recommended by the BEAST program.

The data were evaluated by using the Akaike information criterion in Tracer 1.6 (*5*). The sequences of 13 H5N6 isolates were deposited in the Global Initiative on Sharing Avian Influenza Data database (http://www.gisaid.org) under accession nos. EPI1718935-EPI1719038.

Chicken Test

Intravenous Pathogenicity Index (IVPI)

The pathogenicity of the representative A(H5N6) virus was determined in chicken by means of the IVPI test in accordance with the IVPI protocol of the World Organisation for Animal Health (*6*). Ten 6-week-old SPF chickens were inoculated with 0.2 mL of a 1:10 dilution of $10^{7.0}$ EID_{50}/mL of the virus through the intravenous route, examine the chickens for clinical signs at intervals of 24 hours over a ten-day period (*6*,*7*).

Duck Test

Eight ducks of 3-week-old were intranasally inoculated with 0.1 mL of $10^{6.0}$ EID_{50}/mL of WS/XJ/1/2020(H5N6). At 24 h p.i., three naive ducks from the same flock were co-housed with the infected ducks to monitor the transmission of the A(H5N6) virus (contact group). The ducks were observed for clinical signs daily over 14 days.

Three birds in the infection group were euthanized randomly on day 3 p.i., and the brain, lungs, kidneys, spleen, bursa, thymus, trachea, cecal tonsil, and pancreas were collected for titrating the virus present in these organs in SPF CEEs. Oropharynxgeal and cloacal swabs were collected from the infection and contact groups on days 3, 5, 7, and 9 p.i. and used for titrating the virus in SPF CEEs. On days 14 p.i., serum samples were collected from the surviving birds for detecting homologous hemagglutination-inhibiting antibodies (*6*,*8*).

Mouse Test

To investigate the virulence of WS/XJ/1/2020(H5N6) in mice, six groups of 6-week-old female BALB/c mice (five mice per group) were lightly anesthetized with dry ice. The mice were then separately inoculated intranasally with $10^{1.0}$ to $10^{6.0}$ EID_{50} of the virus in a volume of 50 μL. The control group (five mice) was mock-infected with phosphate-buffered saline. Each group was monitored daily for weight loss and mortality for 14 days. In addition, another group of three mice was inoculated intranasally with $10^{6.0}$ EID_{50} of the A(H5N6) virus in a volume of 50 μL. These mice were euthanized by dry ice on day 3 p.i., and the brain, lungs, kidneys, spleen, and nasal turbinate were collected for virus titration (*6*).

Antigenic Analysis

The HI assay was used to antigenically characterize the A(H5N6) viruses isolated in swan in Xinjiang, western China in 2020 with the current vaccine candidate, Re-11, A/duck/Guizhou/S4184/2017(H5N6). Antisera were generated in 6-week-old white Leghorn SPF chickens, chickens were subcutaneously vaccinated once with 2.0 ml Freund's-adjuvanted inactivated whole virus vaccines (HA content, 9log2). Sera from vaccinated chickens were collected 4 weeks after vaccination (*9*). The HI test was performed using a 1% SPF chicken red blood cell suspension as previously described (*10*). The HI titer was expressed as the reciprocal of the highest serum dilution in which hemagglutination was inhibited.

References

1. Li Y, Shi J, Zhong G, Deng G, Tian G, Ge J, et al. Continued evolution of H5N1 influenza viruses in wild birds, domestic poultry, and humans in China from 2004 to 2009. J Virol. 2010;84:8389–97. PubMed https://doi.org/10.1128/JVI.00413-10

2. Fan Z, Ci Y, Liu L, Ma Y, Jia Y, Wang D, et al. Phylogenetic and pathogenic analyses of three H5N1 avian influenza viruses (clade 2.3.2.1) isolated from wild birds in Northeast China. Infect Genet Evol. 2015;29:138–45. PubMed https://doi.org/10.1016/j.meegid.2014.11.020

3. Drummond AJ, Rambaut A. BEAST: Bayesian evolutionary analysis by sampling trees. BMC Evol Biol. 2007;7:214. PubMed https://doi.org/10.1186/1471-2148-7-214

4. Akaike H. A new look at statistical model identification. IEEE Trans Automat Contr. 1974;19:716–22. https://doi.org/10.1109/TAC.1974.1100705

5. World Organisation for Animal Health. Avian influenza ((infection with avian influenza viruses). In: Manual of diagnostic tests and vaccines for terrestrial animals. 2019 [cited Mar 10]. https://www.oie.int/fileadmin/Home/eng/Health_standards/tahm/3.03.04_AI.pdf

6. Chen H, Deng G, Li Z, Tian G, Li Y, Jiao P, et al. The evolution of H5N1 influenza viruses in ducks in southern China. Proc Natl Acad Sci U S A. 2004;101:10452–7. PubMed https://doi.org/10.1073/pnas.0403212101

7. Neumann G, Chen H, Gao GF, Shu Y, Kawaoka Y. H5N1 influenza viruses: outbreaks and biological properties. Cell Res. 2010;20:51–61. PubMed https://doi.org/10.1038/cr.2009.124

8. Li C, Yu K, Tian G, Yu D, Liu L, Jing B, et al. Evolution of H9N2 influenza viruses from domestic poultry in Mainland China. Virology. 2005;340:70–83. PubMed https://doi.org/10.1016/j.virol.2005.06.025

9. Zeng X, Deng G, Liu L, Li Y, Shi J, Chen P, et al. Protective efficacy of the inactivated H5N1 influenza vaccine Re-6 against different clades of H5N1 viruses isolated in China and the Democratic People's Republic of Korea. Avian Dis. 2016;60(Suppl):238–40. PubMed https://doi.org/10.1637/11178-051915-ResNote

10. Edwards S. OIE laboratory standards for avian influenza. Dev Biol (Basel). 2006;124:159–62. PubMed

Appendix Table 1. The closest relatives to WS/XJ/1/2020(H5N6) in the GISAID database.

Segment	Position	Homology of 13 H5N6 viruses (%)	Closest relatives	Segment ID (EPI_ISL)	Homology (%)
HA	29–1729	99.5–100	A/Env/Guangdong/C17285752-QY/2017(H5N6)	340844	98.8
NA	19–1409	99.9–100	A/Env/Guangdong/C17285752-QY/2017(H5N6)	278026	99.3
PB2	28–2307	99.9–100	A/Duck/Guangdong/PO17281256/MZH/2017(H5N6)	340789	98.9
PB1	25–2298	99.8–100	A/Env/Guangdong/Foshan/C182750085/2018(H5N6)	340824	98.7
PA	25–2175	100	A/Env/Guangdong/Jieyang/C18289059/2018(H5N6)	340825	99.4
NP	46–1542	99.9–100	A/Env/Guangdong/C17285753/QY/2017(H5N6)	340845	98.6
M	26–1007	99.9–100	A/Env/Guangdong/C17285752/QY/2017(H5N6)	340844	99.2
NS	27–855	99.8–100	A/chicken/Nghe_An/01VTC/2018(H5N6)	389022	99.4

Appendix Table 2. Antigenic analysis by chicken sera for clade 2.3.4.4 H5 HPAIV.

Virus	Cross-reactive HI antibody titers of chicken antiserum	
	H5N1 vaccine seed virus Re-11	WS/XJ/1/2020(H5N6)
H5N1 vaccine seed virus Re-11*	512	256
WS/XJ/1/2020(H5N6)	128	1024

*H5 vaccine seed virus Re-11 bears the HA gene from DK/GZ/S4184/17 (H5N6) virus.

A

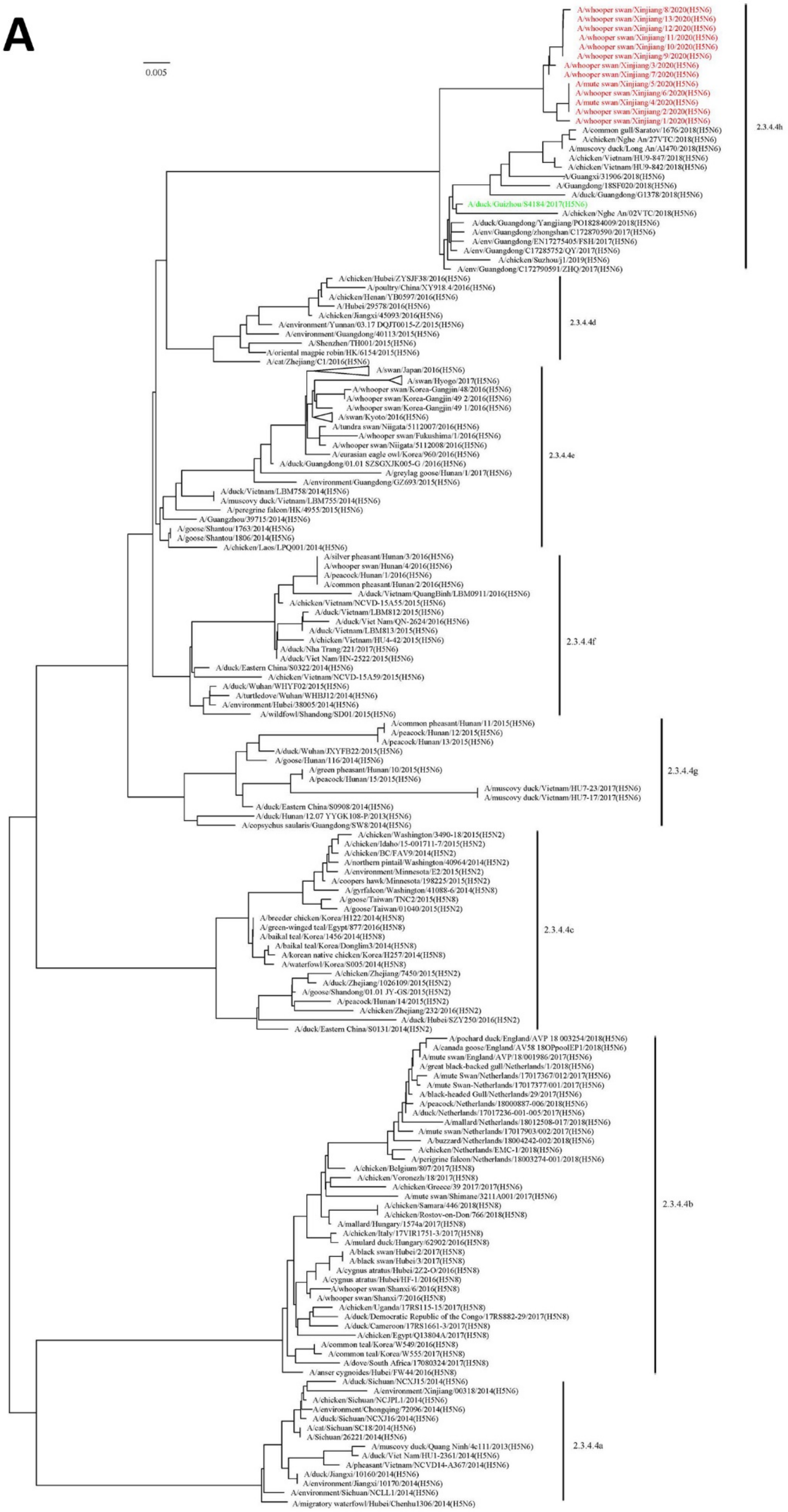

0.005
A/whooper swan/Xinjiang/8/2020(H5N6)
A/whooper swan/Xinjiang/13/2020(H5N6)
A/whooper swan/Xinjiang/12/2020(H5N6)
A/whooper swan/Xinjiang/11/2020(H5N6)
A/whooper swan/Xinjiang/10/2020(H5N6)
A/whooper swan/Xinjiang/9/2020(H5N6)
A/whooper swan/Xinjiang/3/2020(H5N6)
A/whooper swan/Xinjiang/7/2020(H5N6)
A/mute swan/Xinjiang/5/2020(H5N6)
A/whooper swan/Xinjiang/6/2020(H5N6)
A/mute swan/Xinjiang/4/2020(H5N6)
A/whooper swan/Xinjiang/2/2020(H5N6)
A/whooper swan/Xinjiang/1/2020(H5N6)
A/common gull/Saratov/1676/2018(H5N6)
A/chicken/Nghe An/27VTC/2018(H5N6)
A/muscovy duck/Long An/Al470/2018(H5N6)
A/chicken/Vietnam/HU9-847/2018(H5N6)
A/chicken/Vietnam/HU9-842/2018(H5N6)
A/Guangxi/31906/2018(H5N6)
A/Guangdong/18SF020/2018(H5N6)
A/duck/Guangdong/G1378/2018(H5N6)
A/duck/Guizhou/S4184/2017(H5N6)
A/chicken/Nghe An/02VTC/2018(H5N6)
A/duck/Guangdong/Yangjiang/PO18284009/2018(H5N6)
A/env/Guangdong/zhongshan/C172870590/2017(H5N6)
A/env/Guangdong/EN17275405/FSH/2017(H5N6)
A/env/Guangdong/C17285752/QY/2017(H5N6)
A/chicken/Suzhou/j1/2019(H5N6)
A/env/Guangdong/C172790591/ZHQ/2017(H5N6)
2.3.4.4h
A/chicken/Hubei/ZYSJF38/2016(H5N6)
A/poultry/China/XY918.4/2016(H5N6)
A/chicken/Henan/YB0597/2016(H5N6)
A/Hubei/29578/2016(H5N6)
A/chicken/Jiangxi/45093/2016(H5N6)
A/environment/Yunnan/03.17 DQJT0015-Z/2015(H5N6)
A/environment/Guangdong/40113/2015(H5N6)
A/Shenzhen/TH001/2015(H5N6)
A/oriental magpie robin/HK/6154/2015(H5N6)
A/cat/Zhejiang/C1/2016(H5N6)
2.3.4.4d
A/swan/Japan/2016(H5N6)
A/swan/Hyogo/2017(H5N6)
A/whooper swan/Korea-Gangjin/48/2016(H5N6)
A/whooper swan/Korea-Gangjin/49 2/2016(H5N6)
A/whooper swan/Korea-Gangjin/49 1/2016(H5N6)
A/swan/Kyoto/2016(H5N6)
A/tundra swan/Niigata/5112007/2016(H5N6)
A/whooper swan/Fukushima/1/2016(H5N6)
A/whooper swan/Niigata/5112008/2016(H5N6)
A/eurasian eagle owl/Korea/960/2016(H5N6)
A/duck/Guangdong/01.01 SZSGXJK005-G /2016(H5N6)
A/greylag goose/Hunan/1/2017(H5N6)
A/environment/Guangdong/GZ693/2015(H5N6)
A/duck/Vietnam/LBM758/2014(H5N6)
A/muscovy duck/Vietnam/LBM755/2014(H5N6)
A/peregrine falcon/HK/4955/2015(H5N6)
A/Guangzhou/39715/2014(H5N6)
A/goose/Shantou/1763/2014(H5N6)
A/goose/Shantou/1806/2014(H5N6)
A/chicken/Laos/LPQ001/2014(H5N6)
2.3.4.4e
A/silver pheasant/Hunan/3/2016(H5N6)
A/whooper swan/Hunan/4/2016(H5N6)
A/peacock/Hunan/1/2016(H5N6)
A/common pheasant/Hunan/2/2016(H5N6)
A/duck/Vietnam/QuangBinh/LBM0911/2016(H5N6)
A/chicken/Vietnam/NCVD-15A55/2015(H5N6)
A/duck/Vietnam/LBM812/2015(H5N6)
A/duck/Viet Nam/QN-2624/2016(H5N6)
A/duck/Vietnam/LBM813/2015(H5N6)
A/chicken/Vietnam/HU4-42/2015(H5N6)
A/duck/Nha Trang/221/2017(H5N6)
A/duck/Viet Nam/HN-2522/2015(H5N6)
A/duck/Eastern China/S0322/2014(H5N6)
A/chicken/Vietnam/NCVD-15A59/2015(H5N6)
A/duck/Wuhan/WHYF02/2015(H5N6)
A/turtledove/Wuhan/WHBJ12/2014(H5N6)
A/environment/Hubei/38005/2014(H5N6)
A/wildfowl/Shandong/SD01/2015(H5N6)
2.3.4.4f
A/common pheasant/Hunan/11/2015(H5N6)
A/peacock/Hunan/12/2015(H5N6)
A/peacock/Hunan/13/2015(H5N6)
A/duck/Wuhan/JXYFB22/2015(H5N6)
A/goose/Hunan/116/2014(H5N6)
A/green pheasant/Hunan/10/2015(H5N6)
A/peacock/Hunan/15/2015(H5N6)
A/muscovy duck/Vietnam/HU7-23/2017(H5N6)
A/muscovy duck/Vietnam/HU7-17/2017(H5N6)
A/duck/Eastern China/S0908/2014(H5N6)
A/duck/Hunan/12.07 YYGK108-P/2013(H5N6)
A/copsychus saularis/Guangdong/SW8/2014(H5N6)
2.3.4.4g
A/chicken/Washington/3490-18/2015(H5N2)
A/chicken/Idaho/15-001711-7/2015(H5N2)
A/chicken/BC/FAV9/2014(H5N2)
A/northern pintail/Washington/40964/2014(H5N2)
A/environment/Minnesota/E2/2015(H5N2)
A/coopers hawk/Minnesota/198225/2015(H5N2)
A/gyrfalcon/Washington/41088-6/2014(H5N8)
A/goose/Taiwan/TNC2/2015(H5N8)
A/goose/Taiwan/01040/2015(H5N2)
A/breeder chicken/Korea/H122/2014(H5N8)
A/green-winged teal/Egypt/877/2016(H5N8)
A/baikal teal/Korea/1456/2014(H5N8)
A/baikal teal/Korea/Donglim3/2014(H5N8)
A/korean native chicken/Korea/H257/2014(H5N8)
A/waterfowl/Korea/S005/2014(H5N8)
A/chicken/Zhejiang/7450/2015(H5N2)
A/duck/Zhejiang/1026109/2015(H5N2)
A/goose/Shandong/01.01 JY-GS/2015(H5N2)
A/peacock/Hunan/14/2015(H5N2)
A/chicken/Zhejiang/232/2016(H5N2)
A/duck/Hubei/SZY250/2016(H5N2)
A/duck/Eastern China/S0131/2014(H5N2)
2.3.4.4c
A/pochard duck/England/AVP 18 003254/2018(H5N6)
A/canada goose/England/AV58 18OPpoolEP1/2018(H5N6)
A/mute swan/England/AVP/18/001986/2017(H5N6)
A/great black-backed gull/Netherlands/1/2018(H5N6)
A/mute Swan/Netherlands/17017367/012/2017(H5N6)
A/mute Swan-Netherlands/17017377/001/2017(H5N6)
A/black-headed Gull/Netherlands/29/2017(H5N6)
A/peacock/Netherlands/18000887-006/2018(H5N6)
A/duck/Netherlands/17017236-001-005/2017(H5N6)
A/mallard/Netherlands/18012508-017/2018(H5N6)
A/mute swan/Netherlands/17017903/002/2017(H5N6)
A/buzzard/Netherlands/18004242-002/2018(H5N6)
A/chicken/Netherlands/EMC-1/2018(H5N6)
A/perigrine falcon/Netherlands/18003274-001/2018(H5N6)
A/chicken/Belgium/807/2017(H5N8)
A/chicken/Voronezh/18/2017(H5N8)
A/chicken/Greece/39 2017/2017(H5N6)
A/mute swan/Shimane/3211A001/2017(H5N6)
A/chicken/Samara/446/2018(H5N8)
A/chicken/Rostov-on-Don/766/2018(H5N8)
A/mallard/Hungary/1574a/2017(H5N8)
A/chicken/Italy/17VIR1751-3/2017(H5N8)
A/mulard duck/Hungary/62902/2016(H5N8)
A/black swan/Hubei/2/2017(H5N8)
A/black swan/Hubei/3/2017(H5N8)
A/cygnus atratus/Hubei/2/2-O/2016(H5N8)
A/cygnus atratus/Hubei/HF-1/2016(H5N8)
A/whooper swan/Shanxi/6/2016(H5N8)
A/whooper swan/Shanxi/7/2016(H5N8)
A/chicken/Uganda/17RS115-15/2017(H5N8)
A/duck/Democratic Republic of the Congo/17RS882-29/2017(H5N8)
A/duck/Cameroon/17RS1661-3/2017(H5N8)
A/chicken/Egypt/Q13804A/2017(H5N8)
A/common teal/Korea/W549/2016(H5N8)
A/common teal/Korea/W555/2017(H5N8)
A/dove/South Africa/17080324/2017(H5N8)
A/anser cygnoides/Hubei/FW44/2016(H5N8)
2.3.4.4b
A/duck/Sichuan/NCXJ15/2014(H5N6)
A/environment/Xinjiang/00318/2014(H5N6)
A/chicken/Sichuan/NCJPL1/2014(H5N6)
A/environment/Chongqing/72096/2014(H5N6)
A/duck/Sichuan/NCXJ16/2014(H5N6)
A/cat/Sichuan/SC18/2014(H5N6)
A/Sichuan/26221/2014(H5N6)
A/muscovy duck/Quang Ninh/4c111/2013(H5N6)
A/duck/Viet Nam/HU1-2361/2014(H5N6)
A/pheasant/Vietnam/NCVD14-A367/2014(H5N6)
A/duck/Jiangxi/10160/2014(H5N6)
A/environment/Jiangxi/10170/2014(H5N6)
A/environment/Sichuan/NCLL1/2014(H5N6)
A/migratory waterfowl/Hubei/Chenhu1306/2014(H5N6)
2.3.4.4a

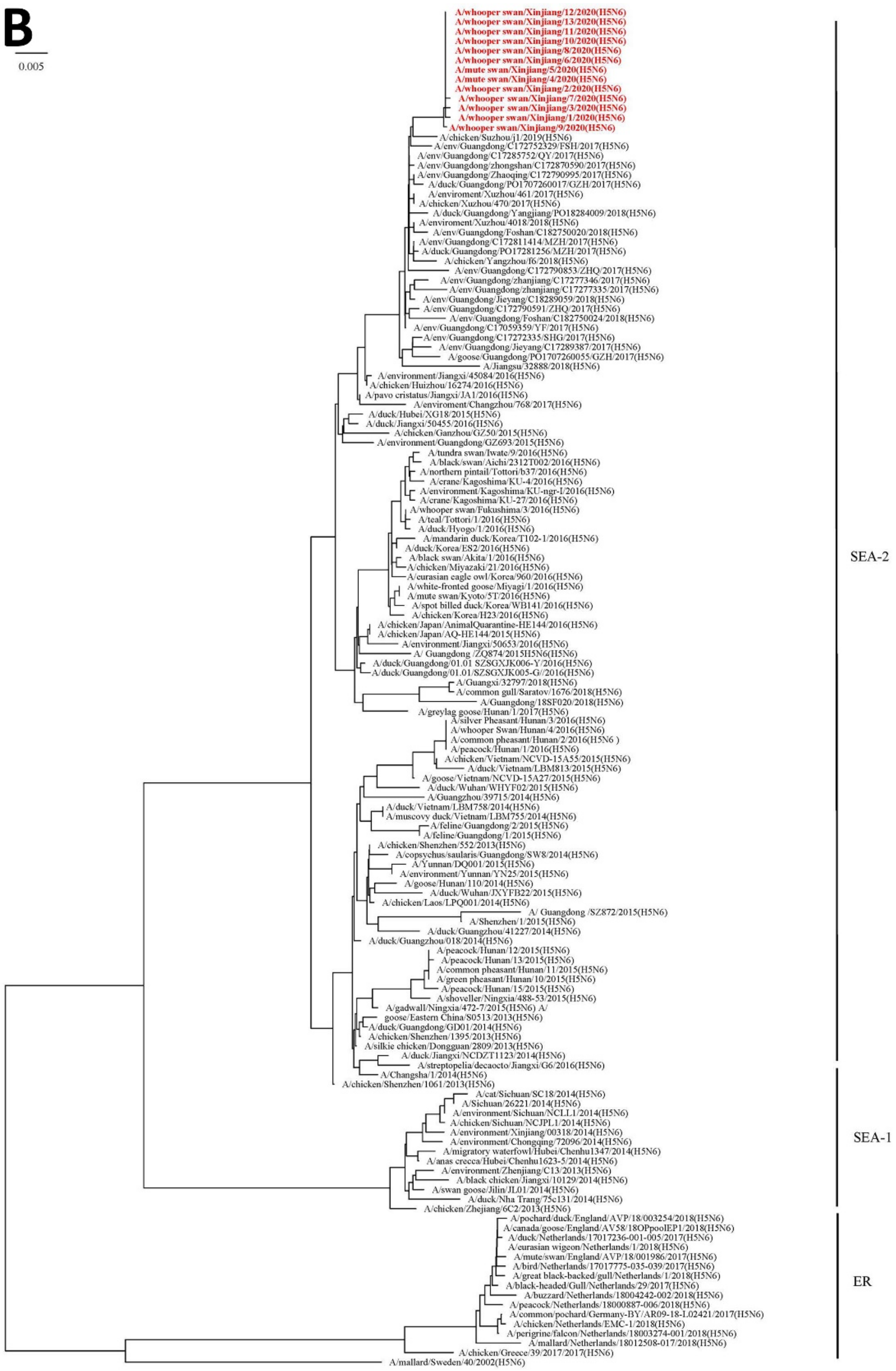
B
0.005
A/whooper swan/Xinjiang/12/2020(H5N6)
A/whooper swan/Xinjiang/13/2020(H5N6)
A/whooper swan/Xinjiang/11/2020(H5N6)
A/whooper swan/Xinjiang/10/2020(H5N6)
A/whooper swan/Xinjiang/8/2020(H5N6)
A/whooper swan/Xinjiang/6/2020(H5N6)
A/mute swan/Xinjiang/5/2020(H5N6)
A/mute swan/Xinjiang/4/2020(H5N6)
A/whooper swan/Xinjiang/2/2020(H5N6)
A/whooper swan/Xinjiang/7/2020(H5N6)
A/whooper swan/Xinjiang/3/2020(H5N6)
A/whooper swan/Xinjiang/1/2020(H5N6)
A/whooper swan/Xinjiang/9/2020(H5N6)
A/chicken/Suzhou/j1/2019(H5N6)
A/env/Guangdong/C172752329/FSH/2017(H5N6)
A/env/Guangdong/C17285752/QY/2017(H5N6)
A/env/Guangdong/zhongshan/C172870590/2017(H5N6)
A/env/Guangdong/Zhaoqing/C172790995/2017(H5N6)
A/duck/Guangdong/PO1707260017/GZH/2017(H5N6)
A/enviroment/Xuzhou/461/2017(H5N6)
A/chicken/Xuzhou/470/2017(H5N6)
A/duck/Guangdong/Yangjiang/PO18284009/2018(H5N6)
A/enviroment/Xuzhou/4018/2018(H5N6)
A/env/Guangdong/Foshan/C182750020/2018(H5N6)
A/env/Guangdong/C172811414/MZH/2017(H5N6)
A/duck/Guangdong/PO17281256/MZH/2017(H5N6)
A/chicken/Yangzhou/f6/2018(H5N6)
A/env/Guangdong/C172790853/ZHQ/2017(H5N6)
A/env/Guangdong/zhanjiang/C17277346/2017(H5N6)
A/env/Guangdong/zhanjiang/C17277335/2017(H5N6)
A/env/Guangdong/Jieyang/C18289059/2018(H5N6)
A/env/Guangdong/C172790591/ZHQ/2017(H5N6)
A/env/Guangdong/Foshan/C182750024/2018(H5N6)
A/env/Guangdong/C17059359/YF/2017(H5N6)
A/env/Guangdong/C17272335/SHG/2017(H5N6)
A/env/Guangdong/Jieyang/C17289387/2017(H5N6)
A/goose/Guangdong/PO1707260055/GZH/2017(H5N6)
A/Jiangsu/32888/2018(H5N6)
A/environment/Jiangxi/45084/2016(H5N6)
A/chicken/Huizhou/16274/2016(H5N6)
A/pavo cristatus/Jiangxi/JA1/2016(H5N6)
A/enviroment/Changzhou/768/2017(H5N6)
A/duck/Hubei/XG18/2015(H5N6)
A/duck/Jiangxi/50455/2016(H5N6)
A/chicken/Ganzhou/GZ50/2015(H5N6)
A/environment/Guangdong/GZ693/2015(H5N6)
A/tundra swan/Iwate/9/2016(H5N6)
A/black/swan/Aichi/2312T002/2016(H5N6)
A/northern pintail/Tottori/b37/2016(H5N6)
A/crane/Kagoshima/KU-4/2016(H5N6)
A/environment/Kagoshima/KU-ngr-I/2016(H5N6)
A/crane/Kagoshima/KU-27/2016(H5N6)
A/whooper swan/Fukushima/3/2016(H5N6)
A/teal/Tottori/1/2016(H5N6)
A/duck/Hyogo/1/2016(H5N6)
A/mandarin duck/Korea/T102-1/2016(H5N6)
A/duck/Korea/ES2/2016(H5N6)
A/black swan/Akita/1/2016(H5N6)
A/chicken/Miyazaki/21/2016(H5N6)
A/eurasian eagle owl/Korea/960/2016(H5N6)
A/white-fronted goose/Miyagi/1/2016(H5N6)
A/mute swan/Kyoto/5T/2016(H5N6)
A/spot billed duck/Korea/WB141/2016(H5N6)
A/chicken/Korea/H23/2016(H5N6)
A/chicken/Japan/AnimalQuarantine-HE144/2016(H5N6)
A/chicken/Japan/AQ-HE144/2015(H5N6)
A/environment/Jiangxi/50653/2016(H5N6)
A/ Guangdong /ZQ874/2015H5N6(H5N6)
A/duck/Guangdong/01.01 SZSGXJK006-Y/2016(H5N6)
A/duck/Guangdong/01.01/SZSGXJK005-G//2016(H5N6)
A/Guangxi/32797/2018(H5N6)
A/common gull/Saratov/1676/2018(H5N6)
A/Guangdong/18SF020/2018(H5N6)
A/greylag goose/Hunan/1/2017(H5N6)
A/silver Pheasant/Hunan/3/2016(H5N6)
A/whooper Swan/Hunan/4/2016(H5N6)
A/common pheasant/Hunan/2/2016(H5N6)
A/peacock/Hunan/1/2016(H5N6)
A/chicken/Vietnam/NCVD-15A55/2015(H5N6)
A/duck/Vietnam/LBM813/2015(H5N6)
A/goose/Vietnam/NCVD-15A27/2015(H5N6)
A/duck/Wuhan/WHYF02/2015(H5N6)
A/Guangzhou/39715/2014(H5N6)
A/duck/Vietnam/LBM758/2014(H5N6)
A/muscovy duck/Vietnam/LBM755/2014(H5N6)
A/feline/Guangdong/2/2015(H5N6)
A/feline/Guangdong/1/2015(H5N6)
A/chicken/Shenzhen/552/2013(H5N6)
A/copsychus/saularis/Guangdong/SW8/2014(H5N6)
A/Yunnan/DQ001/2015(H5N6)
A/environment/Yunnan/YN25/2015(H5N6)
A/goose/Hunan/110/2014(H5N6)
A/duck/Wuhan/JXYFB22/2015(H5N6)
A/chicken/Laos/LPQ001/2014(H5N6)
A/ Guangdong /SZ872/2015(H5N6)
A/Shenzhen/1/2015(H5N6)
A/duck/Guangzhou/41227/2014(H5N6)
A/duck/Guangzhou/018/2014(H5N6)
A/peacock/Hunan/12/2015(H5N6)
A/peacock/Hunan/13/2015(H5N6)
A/common pheasant/Hunan/11/2015(H5N6)
A/green pheasant/Hunan/10/2015(H5N6)
A/peacock/Hunan/15/2015(H5N6)
A/shoveller/Ningxia/488-53/2015(H5N6)
A/gadwall/Ningxia/472-7/2015(H5N6) A/
goose/Eastern China/S0513/2013(H5N6)
A/duck/Guangdong/GD01/2014(H5N6)
A/chicken/Shenzhen/1395/2013(H5N6)
A/silkie chicken/Dongguan/2809/2013(H5N6)
A/duck/Jiangxi/NCDZT1123/2014(H5N6)
A/streptopelia/decaocto/Jiangxi/G6/2016(H5N6)
A/Changsha/1/2014(H5N6)
A/chicken/Shenzhen/1061/2013(H5N6)
A/cat/Sichuan/SC18/2014(H5N6)
A/Sichuan/26221/2014(H5N6)
A/environment/Sichuan/NCLL1/2014(H5N6)
A/chicken/Sichuan/NCJPL1/2014(H5N6)
A/environment/Xinjiang/00318/2014(H5N6)
A/environment/Chongqing/72096/2014(H5N6)
A/migratory waterfowl/Hubei/Chenhu1347/2014(H5N6)
A/anas crecca/Hubei/Chenhu1623-5/2014(H5N6)
A/environment/Zhenjiang/C13/2013(H5N6)
A/black chicken/Jiangxi/10129/2014(H5N6)
A/swan goose/Jilin/JL01/2014(H5N6)
A/duck/Nha Trang/75c131/2014(H5N6)
A/chicken/Zhejiang/6C2/2013(H5N6)
A/pochard/duck/England/AVP/18/003254/2018(H5N6)
A/canada/goose/England/AV58/18OPpoolEP1/2018(H5N6)
A/duck/Netherlands/17017236-001-005/2017(H5N6)
A/eurasian wigeon/Netherlands/1/2018(H5N6)
A/mute/swan/England/AVP/18/001986/2017(H5N6)
A/bird/Netherlands/17017775-035-039/2017(H5N6)
A/great black-backed/gull/Netherlands/1/2018(H5N6)
A/black-headed/Gull/Netherlands/29/2017(H5N6)
A/buzzard/Netherlands/18004242-002/2018(H5N6)
A/peacock/Netherlands/18000887-006/2018(H5N6)
A/common/pochard/Germany-BY/AR09-18-L02421/2017(H5N6)
A/chicken/Netherlands/EMC-1/2018(H5N6)
A/perigrine/falcon/Netherlands/18003274-001/2018(H5N6)
A/mallard/Netherlands/18012508-017/2018(H5N6)
A/chicken/Greece/39/2017/2017(H5N6)
A/mallard/Sweden/40/2002(H5N6)
SEA-2
SEA-1
ER

C

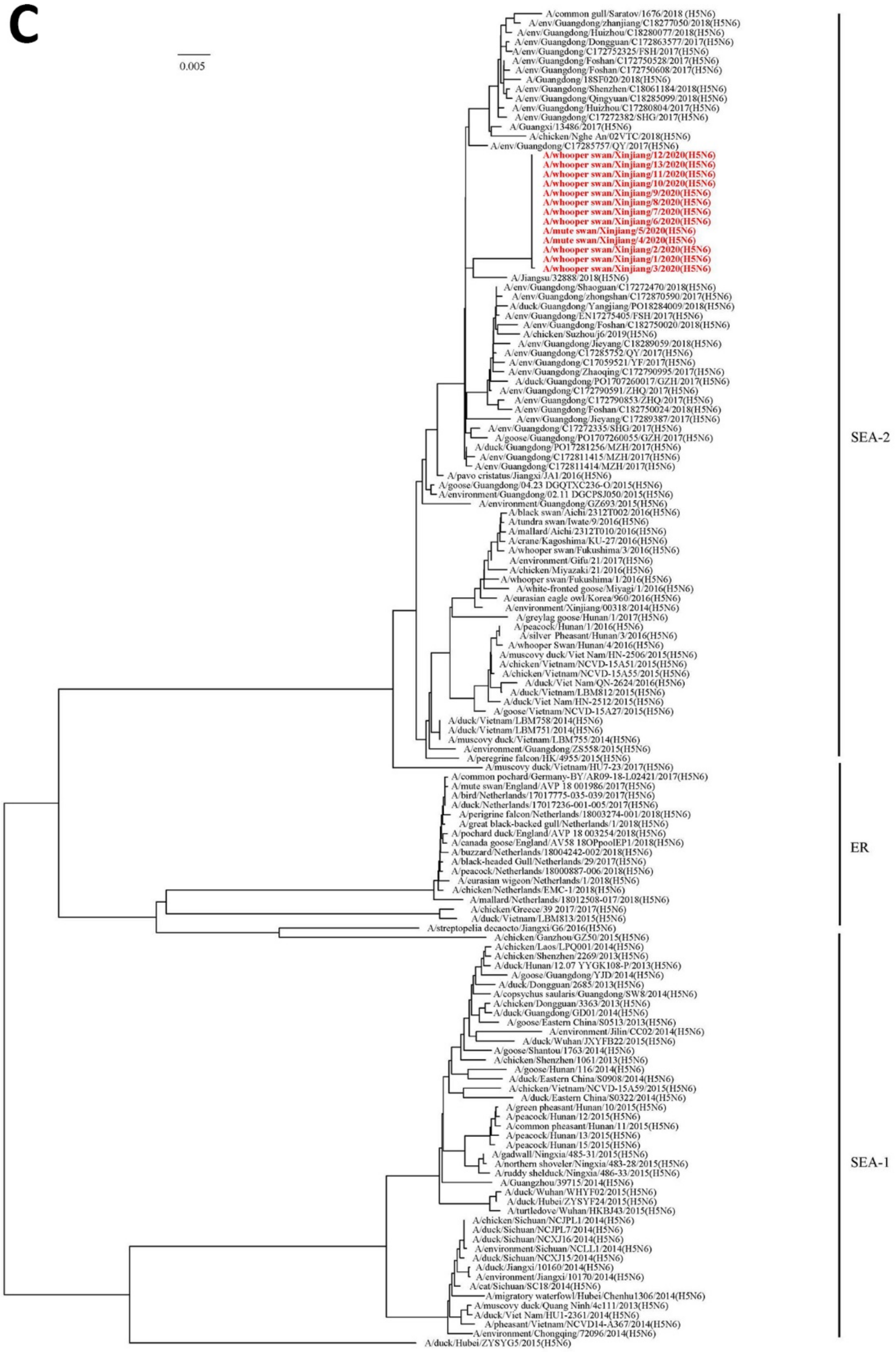
0.005
A/common gull/Saratov/1676/2018 (H5N6)
A/env/Guangdong/zhanjiang/C18277050/2018(H5N6)
A/env/Guangdong/Huizhou/C18280077/2018(H5N6)
A/env/Guangdong/Dongguan/C172863577/2017(H5N6)
A/env/Guangdong/C172752325/FSH/2017(H5N6)
A/env/Guangdong/Foshan/C172750528/2017(H5N6)
A/env/Guangdong/Foshan/C172750608/2017(H5N6)
A/Guangdong/18SF020/2018(H5N6)
A/env/Guangdong/Shenzhen/C18061184/2018(H5N6)
A/env/Guangdong/Qingyuan/C18285099/2018(H5N6)
A/env/Guangdong/Huizhou/C17280804/2017(H5N6)
A/env/Guangdong/C17272382/SHG/2017(H5N6)
A/Guangxi/13486/2017(H5N6)
A/chicken/Nghe An/02VTC/2018(H5N6)
A/env/Guangdong/C17285757/QY/2017(H5N6)
A/whooper swan/Xinjiang/12/2020(H5N6)
A/whooper swan/Xinjiang/13/2020(H5N6)
A/whooper swan/Xinjiang/11/2020(H5N6)
A/whooper swan/Xinjiang/10/2020(H5N6)
A/whooper swan/Xinjiang/9/2020(H5N6)
A/whooper swan/Xinjiang/8/2020(H5N6)
A/whooper swan/Xinjiang/7/2020(H5N6)
A/whooper swan/Xinjiang/6/2020(H5N6)
A/mute swan/Xinjiang/5/2020(H5N6)
A/mute swan/Xinjiang/4/2020(H5N6)
A/whooper swan/Xinjiang/2/2020(H5N6)
A/whooper swan/Xinjiang/1/2020(H5N6)
A/whooper swan/Xinjiang/3/2020(H5N6)
A/Jiangsu/32888/2018(H5N6)
A/env/Guangdong/Shaoguan/C17272470/2018(H5N6)
A/env/Guangdong/zhongshan/C172870590/2017(H5N6)
A/duck/Guangdong/Yangjiang/PO18284009/2018(H5N6)
A/env/Guangdong/EN17275405/FSH/2017(H5N6)
A/env/Guangdong/Foshan/C182750020/2018(H5N6)
A/chicken/Suzhou/j6/2019(H5N6)
A/env/Guangdong/Jieyang/C18289059/2018(H5N6)
A/env/Guangdong/C17285752/QY/2017(H5N6)
A/env/Guangdong/C17059521/YF/2017(H5N6)
A/env/Guangdong/Zhaoqing/C172790995/2017(H5N6)
A/duck/Guangdong/PO1707260017/GZH/2017(H5N6)
A/env/Guangdong/C172790591/ZHQ/2017(H5N6)
A/env/Guangdong/C172790853/ZHQ/2017(H5N6)
A/env/Guangdong/Foshan/C182750024/2018(H5N6)
A/env/Guangdong/Jieyang/C17289387/2017(H5N6)
A/env/Guangdong/C17272335/SHG/2017(H5N6)
A/goose/Guangdong/PO1707260055/GZH/2017(H5N6)
A/duck/Guangdong/PO17281256/MZH/2017(H5N6)
A/env/Guangdong/C172811415/MZH/2017(H5N6)
A/env/Guangdong/C172811414/MZH/2017(H5N6)
A/pavo cristatus/Jiangxi/JA1/2016(H5N6)
A/goose/Guangdong/04.23 DGQTXC236-O/2015(H5N6)
A/environment/Guangdong/02.11 DGCPSJ050/2015(H5N6)
A/environment/Guangdong/GZ693/2015(H5N6)
A/black swan/Aichi/2312T002/2016(H5N6)
A/tundra swan/Iwate/9/2016(H5N6)
A/mallard/Aichi/2312T010/2016(H5N6)
A/crane/Kagoshima/KU-27/2016(H5N6)
A/whooper swan/Fukushima/3/2016(H5N6)
A/environment/Gifu/21/2017(H5N6)
A/chicken/Miyazaki/21/2016(H5N6)
A/whooper swan/Fukushima/1/2016(H5N6)
A/white-fronted goose/Miyagi/1/2016(H5N6)
A/eurasian eagle owl/Korea/960/2016(H5N6)
A/environment/Xinjiang/00318/2014(H5N6)
A/greylag goose/Hunan/1/2017(H5N6)
A/peacock/Hunan/1/2016(H5N6)
A/silver Pheasant/Hunan/3/2016(H5N6)
A/whooper Swan/Hunan/4/2016(H5N6)
A/muscovy duck/Viet Nam/HN-2506/2015(H5N6)
A/chicken/Vietnam/NCVD-15A51/2015(H5N6)
A/chicken/Vietnam/NCVD-15A55/2015(H5N6)
A/duck/Viet Nam/QN-2624/2016(H5N6)
A/duck/Vietnam/LBM812/2015(H5N6)
A/duck/Viet Nam/HN-2512/2015(H5N6)
A/goose/Vietnam/NCVD-15A27/2015(H5N6)
A/duck/Vietnam/LBM758/2014(H5N6)
A/duck/Vietnam/LBM751/2014(H5N6)
A/muscovy duck/Vietnam/LBM755/2014(H5N6)
A/environment/Guangdong/ZS558/2015(H5N6)
A/peregrine falcon/HK/4955/2015(H5N6)
A/muscovy duck/Vietnam/HU7-23/2017(H5N6)
A/common pochard/Germany-BY/AR09-18-L02421/2017(H5N6)
A/mute swan/England/AVP 18 001986/2017(H5N6)
A/bird/Netherlands/17017775-035-039/2017(H5N6)
A/duck/Netherlands/17017236-001-005/2017(H5N6)
A/perigrine falcon/Netherlands/18003274-001/2018(H5N6)
A/great black-backed gull/Netherlands/1/2018(H5N6)
A/pochard duck/England/AVP 18 003254/2018(H5N6)
A/canada goose/England/AV58 18OPpoolEP1/2018(H5N6)
A/buzzard/Netherlands/18004242-002/2018(H5N6)
A/black-headed Gull/Netherlands/29/2017(H5N6)
A/peacock/Netherlands/18000887-006/2018(H5N6)
A/eurasian wigeon/Netherlands/1/2018(H5N6)
A/chicken/Netherlands/EMC-1/2018(H5N6)
A/mallard/Netherlands/18012508-017/2018(H5N6)
A/chicken/Greece/39 2017/2017(H5N6)
A/duck/Vietnam/LBM813/2015(H5N6)
A/streptopelia decaocto/Jiangxi/G6/2016(H5N6)
A/chicken/Ganzhou/GZ50/2015(H5N6)
A/chicken/Laos/LPQ001/2014(H5N6)
A/chicken/Shenzhen/2269/2013(H5N6)
A/duck/Hunan/12.07 YYGK108-P/2013(H5N6)
A/goose/Guangdong/YJD/2014(H5N6)
A/duck/Dongguan/2685/2013(H5N6)
A/copsychus saularis/Guangdong/SW8/2014(H5N6)
A/chicken/Dongguan/3363/2013(H5N6)
A/duck/Guangdong/GD01/2014(H5N6)
A/goose/Eastern China/S0513/2013(H5N6)
A/environment/Jilin/CC02/2014(H5N6)
A/duck/Wuhan/JXYFB22/2015(H5N6)
A/goose/Shantou/1763/2014(H5N6)
A/chicken/Shenzhen/1061/2013(H5N6)
A/goose/Hunan/116/2014(H5N6)
A/duck/Eastern China/S0908/2014(H5N6)
A/chicken/Vietnam/NCVD-15A59/2015(H5N6)
A/duck/Eastern China/S0322/2014(H5N6)
A/green pheasant/Hunan/10/2015(H5N6)
A/peacock/Hunan/12/2015(H5N6)
A/common pheasant/Hunan/11/2015(H5N6)
A/peacock/Hunan/13/2015(H5N6)
A/peacock/Hunan/15/2015(H5N6)
A/gadwall/Ningxia/485-31/2015(H5N6)
A/northern shoveler/Ningxia/483-28/2015(H5N6)
A/ruddy shelduck/Ningxia/486-33/2015(H5N6)
A/Guangzhou/39715/2014(H5N6)
A/duck/Wuhan/WHYF02/2015(H5N6)
A/duck/Hubei/ZYSYF24/2015(H5N6)
A/turtledove/Wuhan/HKBJ43/2015(H5N6)
A/chicken/Sichuan/NCJPL1/2014(H5N6)
A/duck/Sichuan/NCJPL7/2014(H5N6)
A/duck/Sichuan/NCXJ16/2014(H5N6)
A/environment/Sichuan/NCLL1/2014(H5N6)
A/duck/Sichuan/NCXJ15/2014(H5N6)
A/duck/Jiangxi/10160/2014(H5N6)
A/environment/Jiangxi/10170/2014(H5N6)
A/cat/Sichuan/SC18/2014(H5N6)
A/migratory waterfowl/Hubei/Chenhu1306/2014(H5N6)
A/muscovy duck/Quang Ninh/4c111/2013(H5N6)
A/duck/Viet Nam/HU1-2361/2014(H5N6)
A/pheasant/Vietnam/NCVD14-A367/2014(H5N6)
A/environment/Chongqing/72096/2014(H5N6)
A/duck/Hubei/ZYSYG5/2015(H5N6)
SEA-2
ER
SEA-1

D

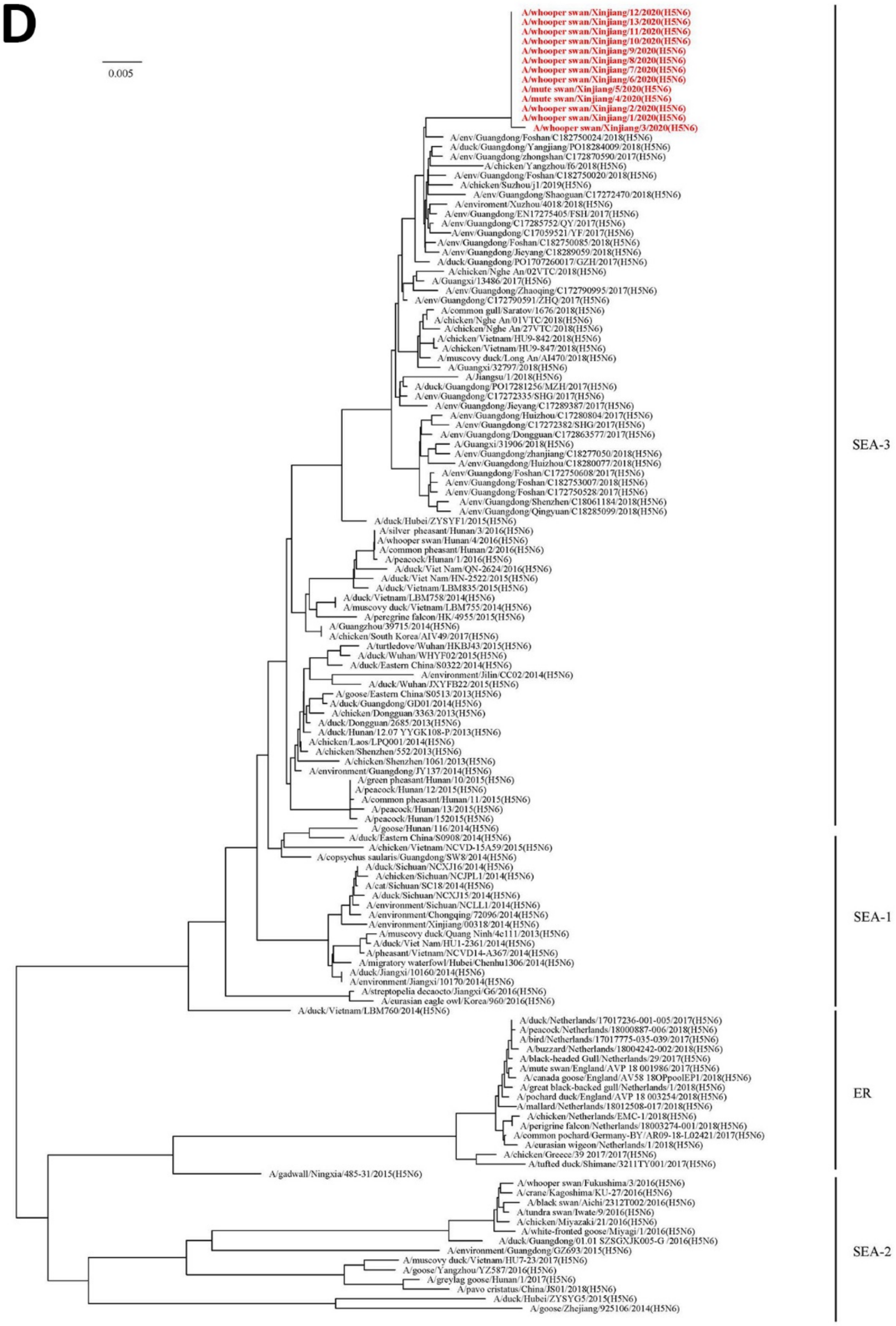

E

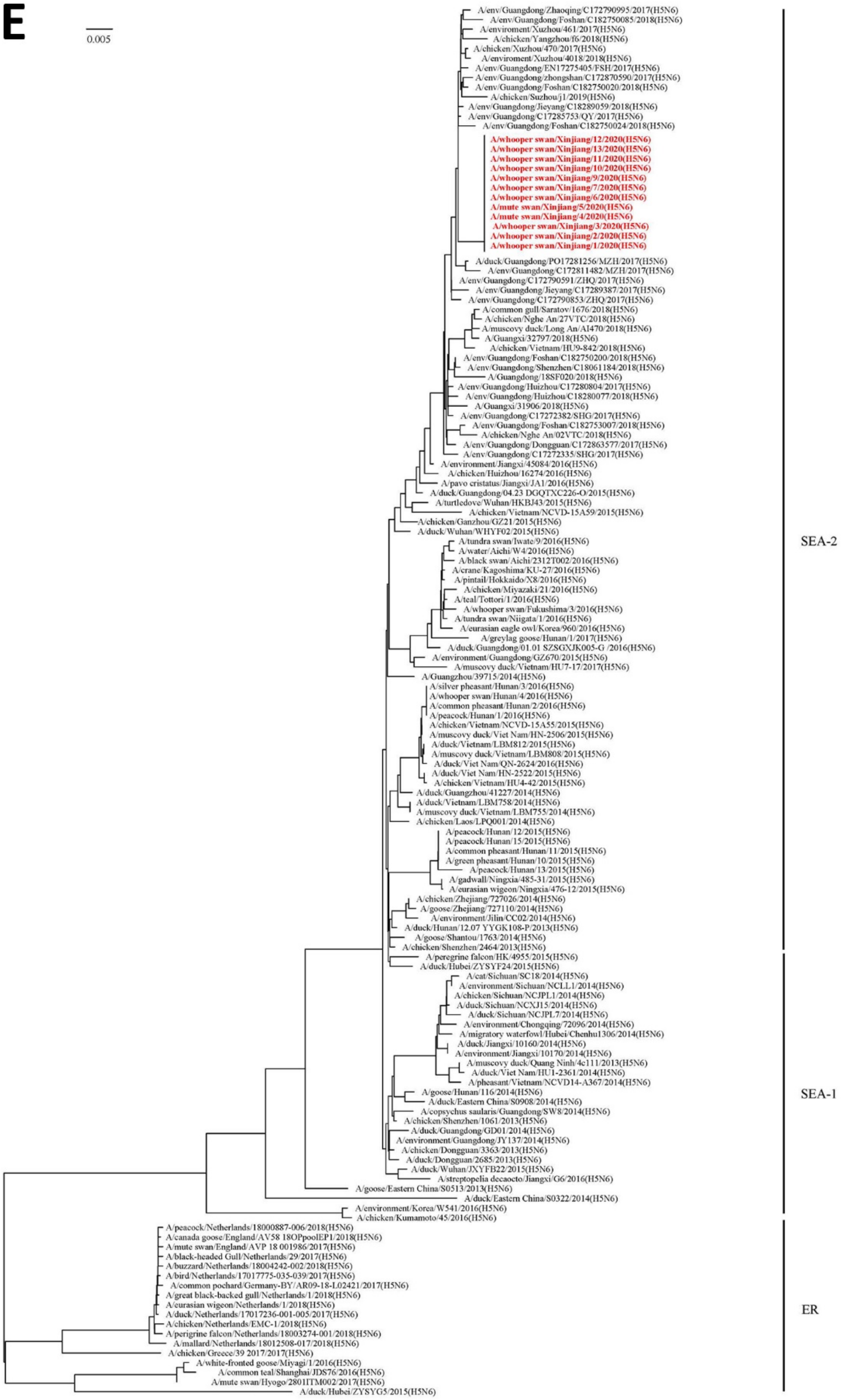
0.005
A/env/Guangdong/Zhaoqing/C172790995/2017(H5N6)
A/env/Guangdong/Foshan/C182750085/2018(H5N6)
A/enviroment/Xuzhou/461/2017(H5N6)
A/chicken/Yangzhou/f6/2018(H5N6)
A/chicken/Xuzhou/470/2017(H5N6)
A/enviroment/Xuzhou/4018/2018(H5N6)
A/env/Guangdong/EN17275405/FSH/2017(H5N6)
A/env/Guangdong/zhongshan/C172870590/2017(H5N6)
A/env/Guangdong/Foshan/C182750020/2018(H5N6)
A/chicken/Suzhou/j1/2019(H5N6)
A/env/Guangdong/Jieyang/C18289059/2018(H5N6)
A/env/Guangdong/C17285753/QY/2017(H5N6)
A/env/Guangdong/Foshan/C182750024/2018(H5N6)
A/whooper swan/Xinjiang/12/2020(H5N6)
A/whooper swan/Xinjiang/13/2020(H5N6)
A/whooper swan/Xinjiang/11/2020(H5N6)
A/whooper swan/Xinjiang/10/2020(H5N6)
A/whooper swan/Xinjiang/9/2020(H5N6)
A/whooper swan/Xinjiang/7/2020(H5N6)
A/whooper swan/Xinjiang/6/2020(H5N6)
A/mute swan/Xinjiang/5/2020(H5N6)
A/mute swan/Xinjiang/4/2020(H5N6)
A/whooper swan/Xinjiang/3/2020(H5N6)
A/whooper swan/Xinjiang/2/2020(H5N6)
A/whooper swan/Xinjiang/1/2020(H5N6)
A/duck/Guangdong/PO17281256/MZH/2017(H5N6)
A/env/Guangdong/C172811482/MZH/2017(H5N6)
A/env/Guangdong/C172790591/ZHQ/2017(H5N6)
A/env/Guangdong/Jieyang/C17289387/2017(H5N6)
A/env/Guangdong/C172790853/ZHQ/2017(H5N6)
A/common gull/Saratov/1676/2018(H5N6)
A/chicken/Nghe An/27VTC/2018(H5N6)
A/muscovy duck/Long An/AI470/2018(H5N6)
A/Guangxi/32797/2018(H5N6)
A/chicken/Vietnam/HU9-842/2018(H5N6)
A/env/Guangdong/Foshan/C182750200/2018(H5N6)
A/env/Guangdong/Shenzhen/C18061184/2018(H5N6)
A/Guangdong/18SF020/2018(H5N6)
A/env/Guangdong/Huizhou/C17280804/2017(H5N6)
A/env/Guangdong/Huizhou/C18280077/2018(H5N6)
A/Guangxi/31906/2018(H5N6)
A/env/Guangdong/C17272382/SHG/2017(H5N6)
A/env/Guangdong/Foshan/C182753007/2018(H5N6)
A/chicken/Nghe An/02VTC/2018(H5N6)
A/env/Guangdong/Dongguan/C172863577/2017(H5N6)
A/env/Guangdong/C17272335/SHG/2017(H5N6)
A/environment/Jiangxi/45084/2016(H5N6)
A/chicken/Huizhou/16274/2016(H5N6)
A/pavo cristatus/Jiangxi/JA1/2016(H5N6)
A/duck/Guangdong/04.23 DGQTXC226-O/2015(H5N6)
A/turtledove/Wuhan/HKBJ43/2015(H5N6)
A/chicken/Vietnam/NCVD-15A59/2015(H5N6)
A/chicken/Ganzhou/GZ21/2015(H5N6)
A/duck/Wuhan/WHYF02/2015(H5N6)
A/tundra swan/Iwate/9/2016(H5N6)
A/water/Aichi/W4/2016(H5N6)
A/black swan/Aichi/2312T002/2016(H5N6)
A/crane/Kagoshima/KU-27/2016(H5N6)
A/pintail/Hokkaido/X8/2016(H5N6)
A/chicken/Miyazaki/21/2016(H5N6)
A/teal/Tottori/1/2016(H5N6)
A/whooper swan/Fukushima/3/2016(H5N6)
A/tundra swan/Niigata/1/2016(H5N6)
A/eurasian eagle owl/Korea/960/2016(H5N6)
A/greylag goose/Hunan/1/2017(H5N6)
A/duck/Guangdong/01.01 SZSGXJK005-G /2016(H5N6)
A/environment/Guangdong/GZ670/2015(H5N6)
A/muscovy duck/Vietnam/HU7-17/2017(H5N6)
A/Guangzhou/39715/2014(H5N6)
A/silver pheasant/Hunan/3/2016(H5N6)
A/whooper swan/Hunan/4/2016(H5N6)
A/common pheasant/Hunan/2/2016(H5N6)
A/peacock/Hunan/1/2016(H5N6)
A/chicken/Vietnam/NCVD-15A55/2015(H5N6)
A/muscovy duck/Viet Nam/HN-2506/2015(H5N6)
A/duck/Vietnam/LBM812/2015(H5N6)
A/muscovy duck/Vietnam/LBM808/2015(H5N6)
A/duck/Viet Nam/QN-2624/2016(H5N6)
A/duck/Viet Nam/HN-2522/2015(H5N6)
A/chicken/Vietnam/HU4-42/2015(H5N6)
A/duck/Guangzhou/41227/2014(H5N6)
A/duck/Vietnam/LBM758/2014(H5N6)
A/muscovy duck/Vietnam/LBM755/2014(H5N6)
A/chicken/Laos/LPQ001/2014(H5N6)
A/peacock/Hunan/12/2015(H5N6)
A/peacock/Hunan/15/2015(H5N6)
A/common pheasant/Hunan/11/2015(H5N6)
A/green pheasant/Hunan/10/2015(H5N6)
A/peacock/Hunan/13/2015(H5N6)
A/gadwall/Ningxia/485-31/2015(H5N6)
A/eurasian wigeon/Ningxia/476-12/2015(H5N6)
A/chicken/Zhejiang/727026/2014(H5N6)
A/goose/Zhejiang/727110/2014(H5N6)
A/environment/Jilin/CC02/2014(H5N6)
A/duck/Hunan/12.07 YYGK108-P/2013(H5N6)
A/goose/Shantou/1763/2014(H5N6)
A/chicken/Shenzhen/2464/2013(H5N6)
A/peregrine falcon/HK/4955/2015(H5N6)
A/duck/Hubei/ZYSYF24/2015(H5N6)
A/cat/Sichuan/SC18/2014(H5N6)
A/environment/Sichuan/NCLL1/2014(H5N6)
A/chicken/Sichuan/NCJPL1/2014(H5N6)
A/duck/Sichuan/NCXJ15/2014(H5N6)
A/duck/Sichuan/NCJPL7/2014(H5N6)
A/environment/Chongqing/72096/2014(H5N6)
A/migratory waterfowl/Hubei/Chenhu1306/2014(H5N6)
A/duck/Jiangxi/10160/2014(H5N6)
A/environment/Jiangxi/10170/2014(H5N6)
A/muscovy duck/Quang Ninh/4c111/2013(H5N6)
A/duck/Viet Nam/HU1-2361/2014(H5N6)
A/pheasant/Vietnam/NCVD14-A367/2014(H5N6)
A/goose/Hunan/116/2014(H5N6)
A/duck/Eastern China/S0908/2014(H5N6)
A/copsychus saularis/Guangdong/SW8/2014(H5N6)
A/chicken/Shenzhen/1061/2013(H5N6)
A/duck/Guangdong/GD01/2014(H5N6)
A/environment/Guangdong/JY137/2014(H5N6)
A/chicken/Dongguan/3363/2013(H5N6)
A/duck/Dongguan/2685/2013(H5N6)
A/duck/Wuhan/JXYFB22/2015(H5N6)
A/streptopelia decaocto/Jiangxi/G6/2016(H5N6)
A/goose/Eastern China/S0513/2013(H5N6)
A/duck/Eastern China/S0322/2014(H5N6)
A/environment/Korea/W541/2016(H5N6)
A/chicken/Kumamoto/45/2016(H5N6)
A/peacock/Netherlands/18000887-006/2018(H5N6)
A/canada goose/England/AV58 18OPpoolEP1/2018(H5N6)
A/mute swan/England/AVP 18 001986/2017(H5N6)
A/black-headed Gull/Netherlands/29/2017(H5N6)
A/buzzard/Netherlands/18004242-002/2018(H5N6)
A/bird/Netherlands/17017775-035-039/2017(H5N6)
A/common pochard/Germany-BY/AR09-18-L02421/2017(H5N6)
A/great black-backed gull/Netherlands/1/2018(H5N6)
A/eurasian wigeon/Netherlands/1/2018(H5N6)
A/duck/Netherlands/17017236-001-005/2017(H5N6)
A/chicken/Netherlands/EMC-1/2018(H5N6)
A/perigrine falcon/Netherlands/18003274-001/2018(H5N6)
A/mallard/Netherlands/18012508-017/2018(H5N6)
A/chicken/Greece/39 2017/2017(H5N6)
A/white-fronted goose/Miyagi/1/2016(H5N6)
A/common teal/Shanghai/JDS76/2016(H5N6)
A/mute swan/Hyogo/2801ITM002/2017(H5N6)
A/duck/Hubei/ZYSYG5/2015(H5N6)
SEA-2
SEA-1
ER

F

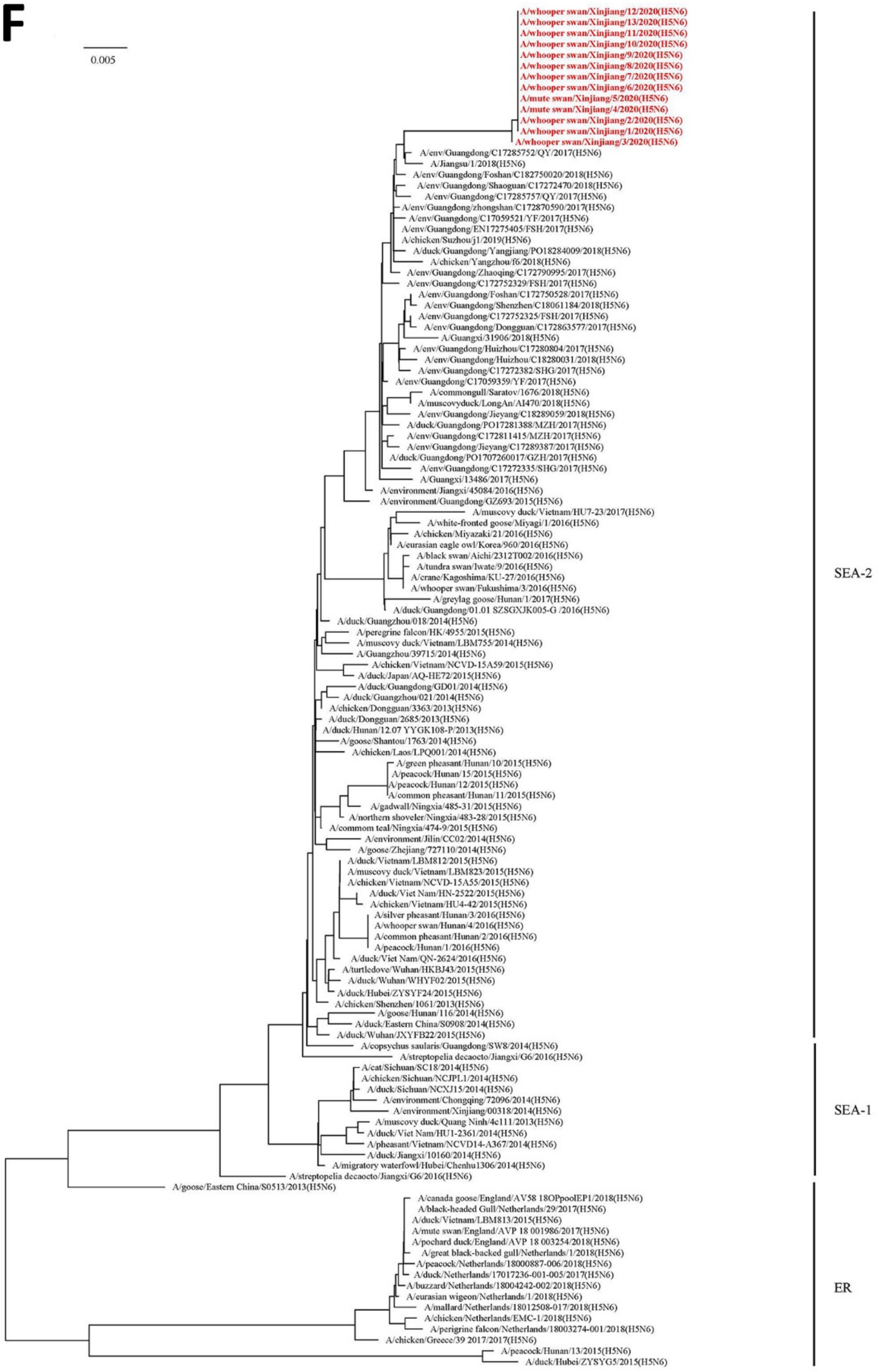

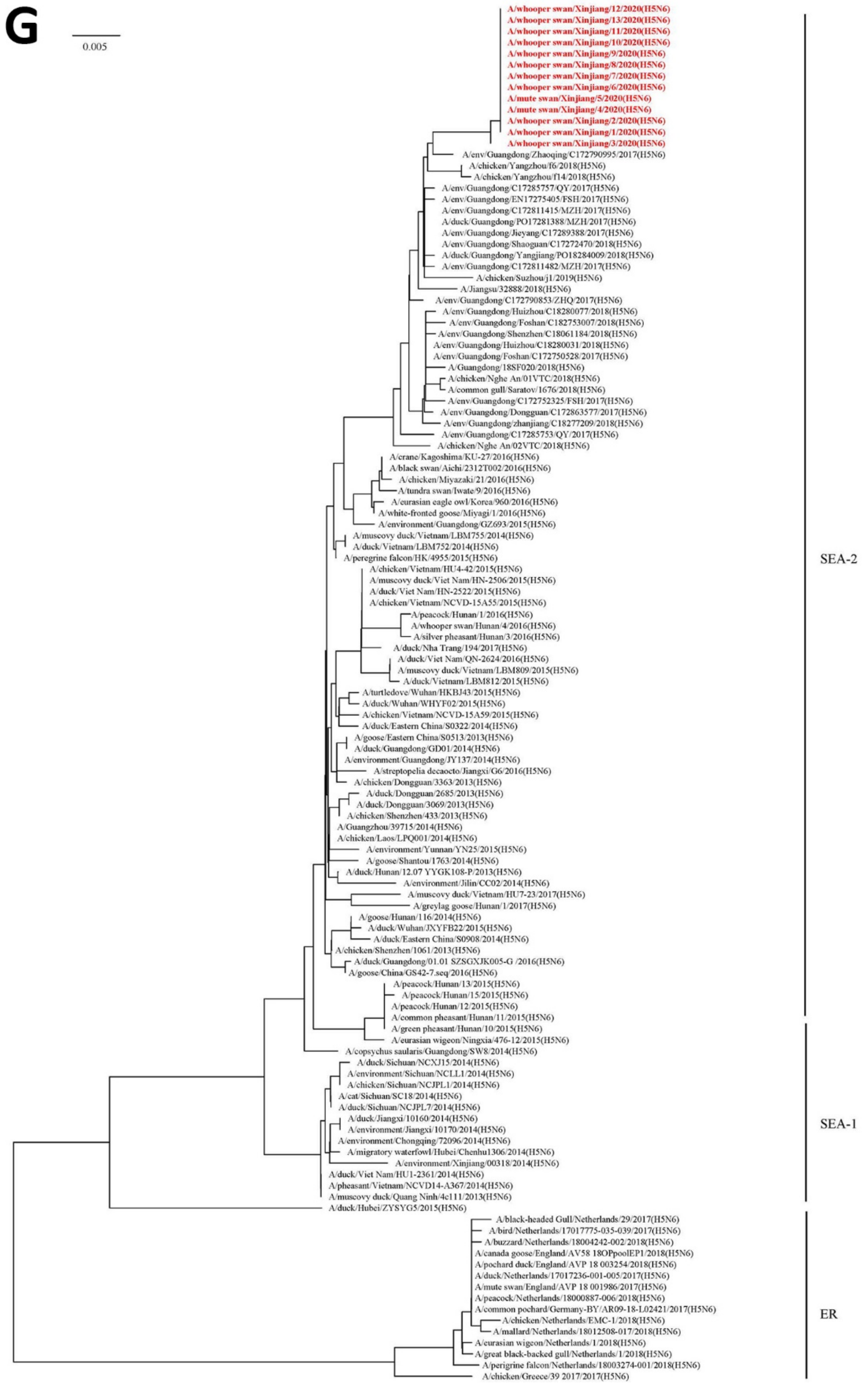
G
0.005
A/whooper swan/Xinjiang/12/2020(H5N6)
A/whooper swan/Xinjiang/13/2020(H5N6)
A/whooper swan/Xinjiang/11/2020(H5N6)
A/whooper swan/Xinjiang/10/2020(H5N6)
A/whooper swan/Xinjiang/9/2020(H5N6)
A/whooper swan/Xinjiang/8/2020(H5N6)
A/whooper swan/Xinjiang/7/2020(H5N6)
A/whooper swan/Xinjiang/6/2020(H5N6)
A/mute swan/Xinjiang/5/2020(H5N6)
A/mute swan/Xinjiang/4/2020(H5N6)
A/whooper swan/Xinjiang/2/2020(H5N6)
A/whooper swan/Xinjiang/1/2020(H5N6)
A/whooper swan/Xinjiang/3/2020(H5N6)
A/env/Guangdong/Zhaoqing/C172790995/2017(H5N6)
A/chicken/Yangzhou/f6/2018(H5N6)
A/chicken/Yangzhou/f14/2018(H5N6)
A/env/Guangdong/C17285757/QY/2017(H5N6)
A/env/Guangdong/EN17275405/FSH/2017(H5N6)
A/env/Guangdong/C172811415/MZH/2017(H5N6)
A/duck/Guangdong/PO17281388/MZH/2017(H5N6)
A/env/Guangdong/Jieyang/C17289388/2017(H5N6)
A/env/Guangdong/Shaoguan/C17272470/2018(H5N6)
A/duck/Guangdong/Yangjiang/PO18284009/2018(H5N6)
A/env/Guangdong/C172811482/MZH/2017(H5N6)
A/chicken/Suzhou/j1/2019(H5N6)
A/Jiangsu/32888/2018(H5N6)
A/env/Guangdong/C172790853/ZHQ/2017(H5N6)
A/env/Guangdong/Huizhou/C18280077/2018(H5N6)
A/env/Guangdong/Foshan/C182753007/2018(H5N6)
A/env/Guangdong/Shenzhen/C18061184/2018(H5N6)
A/env/Guangdong/Huizhou/C18280031/2018(H5N6)
A/env/Guangdong/Foshan/C172750528/2017(H5N6)
A/Guangdong/18SF020/2018(H5N6)
A/chicken/Nghe An/01VTC/2018(H5N6)
A/common gull/Saratov/1676/2018(H5N6)
A/env/Guangdong/C172752325/FSH/2017(H5N6)
A/env/Guangdong/Dongguan/C172863577/2017(H5N6)
A/env/Guangdong/zhanjiang/C18277209/2018(H5N6)
A/env/Guangdong/C17285753/QY/2017(H5N6)
A/chicken/Nghe An/02VTC/2018(H5N6)
A/crane/Kagoshima/KU-27/2016(H5N6)
A/black swan/Aichi/2312T002/2016(H5N6)
A/chicken/Miyazaki/21/2016(H5N6)
A/tundra swan/Iwate/9/2016(H5N6)
A/eurasian eagle owl/Korea/960/2016(H5N6)
A/white-fronted goose/Miyagi/1/2016(H5N6)
A/environment/Guangdong/GZ693/2015(H5N6)
A/muscovy duck/Vietnam/LBM755/2014(H5N6)
A/duck/Vietnam/LBM752/2014(H5N6)
A/peregrine falcon/HK/4955/2015(H5N6)
A/chicken/Vietnam/HU4-42/2015(H5N6)
A/muscovy duck/Viet Nam/HN-2506/2015(H5N6)
A/duck/Viet Nam/HN-2522/2015(H5N6)
A/chicken/Vietnam/NCVD-15A55/2015(H5N6)
A/peacock/Hunan/1/2016(H5N6)
A/whooper swan/Hunan/4/2016(H5N6)
A/silver pheasant/Hunan/3/2016(H5N6)
A/duck/Nha Trang/194/2017(H5N6)
A/duck/Viet Nam/QN-2624/2016(H5N6)
A/muscovy duck/Vietnam/LBM809/2015(H5N6)
A/duck/Vietnam/LBM812/2015(H5N6)
A/turtledove/Wuhan/HKBJ43/2015(H5N6)
A/duck/Wuhan/WHYF02/2015(H5N6)
A/chicken/Vietnam/NCVD-15A59/2015(H5N6)
A/duck/Eastern China/S0322/2014(H5N6)
A/goose/Eastern China/S0513/2013(H5N6)
A/duck/Guangdong/GD01/2014(H5N6)
A/environment/Guangdong/JY137/2014(H5N6)
A/streptopelia decaocto/Jiangxi/G6/2016(H5N6)
A/chicken/Dongguan/3363/2013(H5N6)
A/duck/Dongguan/2685/2013(H5N6)
A/duck/Dongguan/3069/2013(H5N6)
A/chicken/Shenzhen/433/2013(H5N6)
A/Guangzhou/39715/2014(H5N6)
A/chicken/Laos/LPQ001/2014(H5N6)
A/environment/Yunnan/YN25/2015(H5N6)
A/goose/Shantou/1763/2014(H5N6)
A/duck/Hunan/12.07 YYGK108-P/2013(H5N6)
A/environment/Jilin/CC02/2014(H5N6)
A/muscovy duck/Vietnam/HU7-23/2017(H5N6)
A/greylag goose/Hunan/1/2017(H5N6)
A/goose/Hunan/116/2014(H5N6)
A/duck/Wuhan/JXYFB22/2015(H5N6)
A/duck/Eastern China/S0908/2014(H5N6)
A/chicken/Shenzhen/1061/2013(H5N6)
A/duck/Guangdong/01.01 SZSGXJK005-G /2016(H5N6)
A/goose/China/GS42-7.seq/2016(H5N6)
A/peacock/Hunan/13/2015(H5N6)
A/peacock/Hunan/15/2015(H5N6)
A/peacock/Hunan/12/2015(H5N6)
A/common pheasant/Hunan/11/2015(H5N6)
A/green pheasant/Hunan/10/2015(H5N6)
A/eurasian wigeon/Ningxia/476-12/2015(H5N6)
A/copsychus saularis/Guangdong/SW8/2014(H5N6)
A/duck/Sichuan/NCXJ15/2014(H5N6)
A/environment/Sichuan/NCLL1/2014(H5N6)
A/chicken/Sichuan/NCJPL1/2014(H5N6)
A/cat/Sichuan/SC18/2014(H5N6)
A/duck/Sichuan/NCJPL7/2014(H5N6)
A/duck/Jiangxi/10160/2014(H5N6)
A/environment/Jiangxi/10170/2014(H5N6)
A/environment/Chongqing/72096/2014(H5N6)
A/migratory waterfowl/Hubei/Chenhu1306/2014(H5N6)
A/environment/Xinjiang/00318/2014(H5N6)
A/duck/Viet Nam/HU1-2361/2014(H5N6)
A/pheasant/Vietnam/NCVD14-A367/2014(H5N6)
A/muscovy duck/Quang Ninh/4c111/2013(H5N6)
A/duck/Hubei/ZYSYG5/2015(H5N6)
A/black-headed Gull/Netherlands/29/2017(H5N6)
A/bird/Netherlands/17017775-035-039/2017(H5N6)
A/buzzard/Netherlands/18004242-002/2018(H5N6)
A/canada goose/England/AV58 18OPpoolEP1/2018(H5N6)
A/pochard duck/England/AVP 18 003254/2018(H5N6)
A/duck/Netherlands/17017236-001-005/2017(H5N6)
A/mute swan/England/AVP 18 001986/2017(H5N6)
A/peacock/Netherlands/18000887-006/2018(H5N6)
A/common pochard/Germany-BY/AR09-18-L02421/2017(H5N6)
A/chicken/Netherlands/EMC-1/2018(H5N6)
A/mallard/Netherlands/18012508-017/2018(H5N6)
A/eurasian wigeon/Netherlands/1/2018(H5N6)
A/great black-backed gull/Netherlands/1/2018(H5N6)
A/perigrine falcon/Netherlands/18003274-001/2018(H5N6)
A/chicken/Greece/39 2017/2017(H5N6)
SEA-2
SEA-1
ER

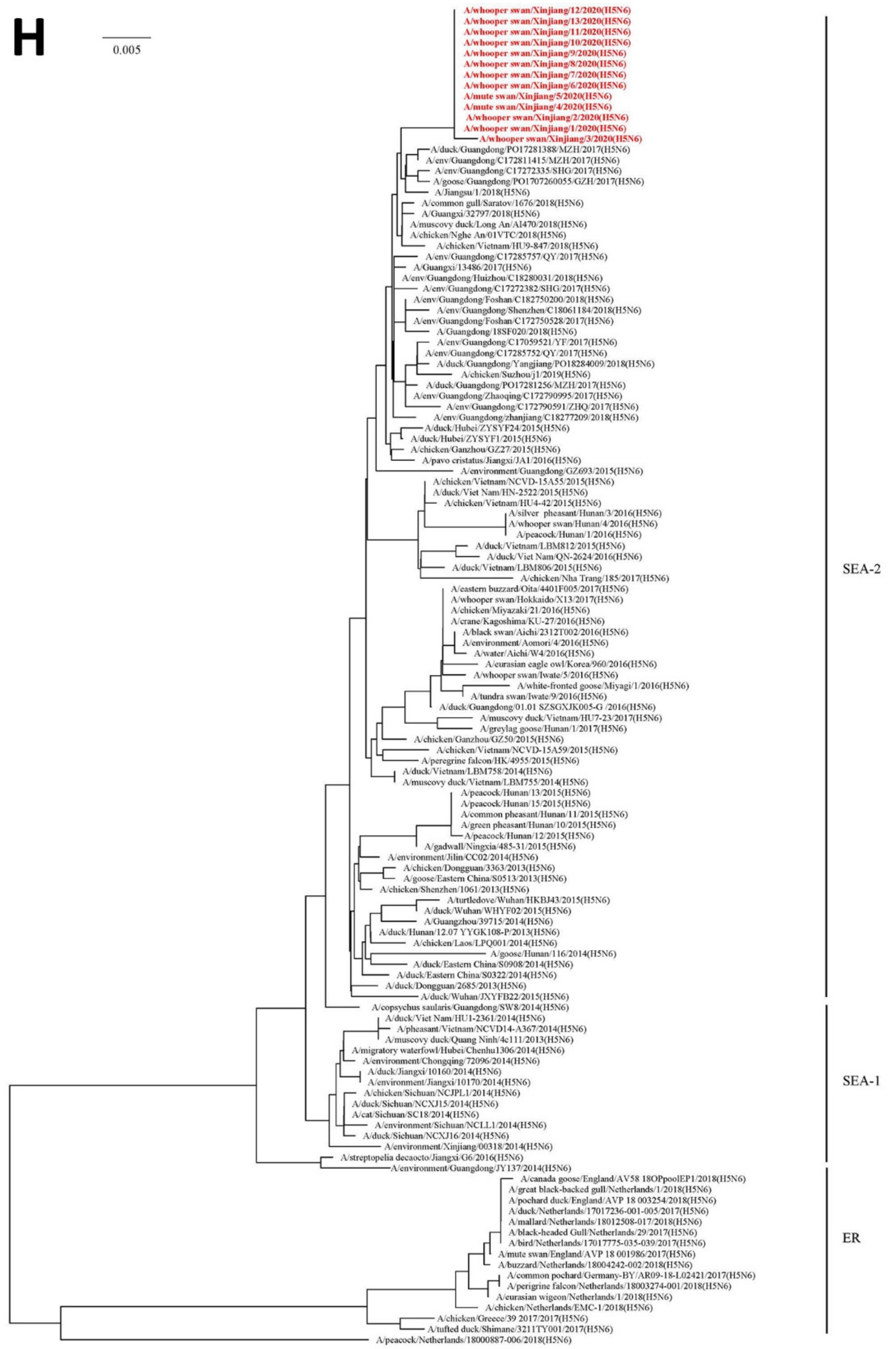

Appendix Figure. Phylogenetic analysis of SW/XJ/2020(H5N6). The neighbor joining trees of SW/XJ/2020(H5N6) virus genes were built by using the Mega 6.0.6 ClustalW software package. Segments shown are: hemagglutinin (HA) (A); neuraminidase (NA) (B); polymerase basic (PB2) (C); polymerase basic (PB1) (D); polymerase (PA) (E); nucleoprotein (NP) (F); matrix (M) (G); Nonstructural

protein (NS) (H). The unrooted trees were based on nucleotide positions 29 to 1701 for HA, 29 to 1409 for N6 NA, 28 to 2307 for PB2, 25 to 2298 for PB1, 25 to 2175 for PA, 46 to 1542 for NP, 26 to 1007 for M, and 27 to 855 for NS. The 13 H5N6 from the swans in this study are shown in red and bold, the HA donor of Re-11 vaccine candidate is shown in green and bold in panel A. SEA, Southeast Asia; ER, Europe.
